# Double Electrode Experiments Reveal the Processes Occurring at PEDOT-Coated Neural Electrode Arrays

**DOI:** 10.1021/acsami.4c05204

**Published:** 2024-05-22

**Authors:** Yuanmin Zhang, Yuqi Chen, Sonia Contera, Richard G. Compton

**Affiliations:** † Clarendon Laboratory, Department of Physics, University of Oxford, Parks Road, Oxford OX1 3PU, Great Britain; ‡ Physical and Theoretical Chemistry Laboratory, Department of Chemistry, University of Oxford, South Parks Road, Oxford OX1 3QZ, Great Britain

**Keywords:** poly(3,4-ethylenedioxythiophene):poly(styrenesulfonate) (PEDOT:PSS), poly(3,4-ethylenedioxythiophene):chloride (PEDOT:Cl), electrochemical analysis, cyclic voltammetry (CV), bipotentiostat, tetrode

## Abstract

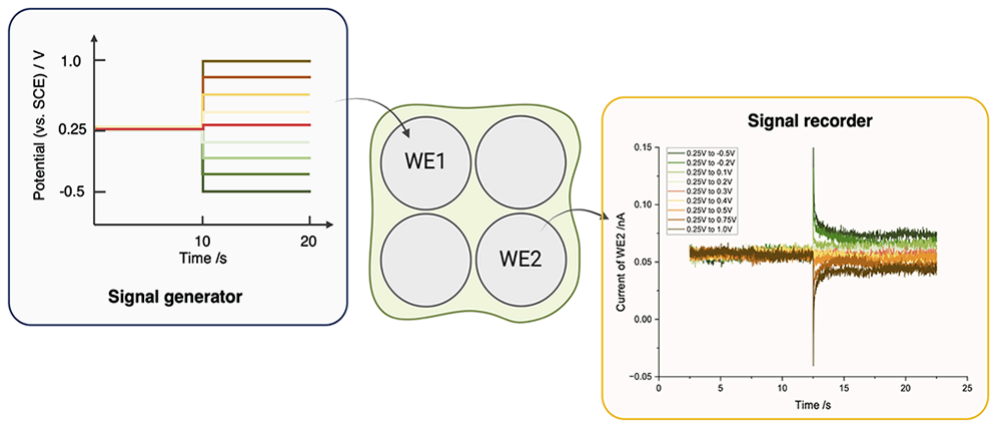

Neural electrodes have recently been developed with surface modifications of conductive polymers, in particular poly(3,4-ethylenedioxythiophene) (PEDOT), and extensively studied for their roles in recording and stimulation, aiming to improve their biocompatibility. In this work, the implications for the design of practical neural sensors are clarified, and systematic procedures for their preparation are reported. In particular, this study introduces the use of in vitro double electrode experiments to mimic the responses of neural electrodes with a focus on signal-recording electrodes modified with PEDOT. Specifically, potential steps on one unmodified electrode in an array are used to identify the responses for PEDOT doped with different anions and compared with that of a bare platinum (Pt) electrode. The response is shown to be related to the rearrangement of ions in solution near the detector electrode resulting from the potential step, with a current transient seen at the detector electrode. A rapid response for PEDOT doped with chloride (ca. 0.04 s) ions was observed and attributed to the fast movement of chloride ions in and out of the polymer film. In contrast, PEDOT doped with poly(styrenesulfonate) (PSS) responds much slower (ca. 2.2 s), and the essential immobility of polyanion constrains the direction of current flow.

## 1 Introduction

Neuronal activity in the central nervous system gives rise to transmembrane currents that can be detected by electrodes in the extracellular medium. These “electrical recordings” are used by neuroscientists to investigate the processes underlying neuronal communication and computation.^1^ Significant research has focused on developing neural recording electrodes to collect and interpret these signals in the brain. Extracellular recording started with a tungsten microwire electrode,^2^ advancing to silicon probes, among which the Michigan array^3,4^ and Utah array^5,6^ are the most widely used. The Michigan array consists of a single or several long “shanks”^4^ with distributed recording sites. The Utah array is a 10 × 10 array of silicon needles on a large silicon base. Subsequent developments include tetrodes, formed by twisting microwires together,^7,8^ and, more recently, flexible polymer electrode arrays such as NeuroGrid^9,10^ and mesh electronics.^11,12^ A summary of the various electrodes is presented in Table 1.

**Table 1:** Summary of Neural Electrodes

Electrode Techniques	Description	Function	Refs
Tungsten microwire electrode	One of the earliest types of neuron electrodes was reported to have a sharpened tip with a submicrometer diameter, enabling it to record signals from small neurons and axons in the mammalian brain.	Recording	(2)
Michigan array	The Michigan array consists of one to several long shanks, with recording sites distributed along each shank. Typically, the shank width is 120 μm, and its thickness ranges from 15 to 50 μm.	Recording and stimulating	(3, 4, 18)
Utah array	The Utah array consists of 100 microelectrodes arranged in a 10 × 10 pattern. These silicon needles typically have a length of a few millimeters, projecting from a silicon base, with dimensions of approximately 4.2 × 4.2 mm.	Recording and stimulation	(5, 6, 16)
Tetrode	Tetrode is formed by twisting four insulated microwires together. The microwire diameter usually ranges from 12 to 25 μm.	Recording	(7, 8)
NeuroGrid	NeuroGrid consists of electrodes on a flexible and soft polymer substrate, which enables surface-level large-scale monitoring of neural activities. The polymer film is typically a few micrometers thick.	Recording	(9, 10)
Mesh electronics	Mesh electronics feature a soft, tissue-like design, with probes approximately the size of neuron soma, interconnected by mesh-like structured nanowires. The implantation process involves syringe injection to ensure minimal invasiveness.	Recording and stimulation	(11, 12)

The signals or spikes recorded by extracellular electrodes are generated by ion flow induced near active neurons,^13^ and, as discussed below, this paper proposes a novel dual electrode approach to mimic and understand the signals and spikes recorded in neural electrodes. Extracellular recordings typically embrace signals from multiple neurons within a proximal range (up to around 140 μm).^1,14^ Hence, a single detection channel can capture signals from various neurons. If the spikes have minimal overlap so that they can be temporally resolved,^15^ then active neurons can be located using triangulation methods by analyzing the amplitudes of the wavefronts from different channels.^8^ Additionally, the distinct shape of each action potential helps in identifying individual neurons.^8,15^ Silicon probes, usually with numerous detection sites (ranging from 8 to 1024 recording sites^1,16,17^), are designed to capture as many signals as possible, and such oversampling can facilitate spike separation and assignment.^1^ However, their relatively large physical size poses challenges, causing tissue damage, particularly when penetrating deep into the brain.^1,16^ For instance, a Michigan array is often approximately 120 μm in width and 15 to 50 μm in thickness,^18^ and a Utah array has 100 silicon needles (around 80–100 μm thick at base^5,6,16^) projecting from a large substrate (4.2 × 4.2 mm).^5^ This penetration can lead to inflammation and glial encapsulation that prevents external signals from reaching the electrode.^1,16,19^ To address these issues, recent developments in electrode technology have focused on creating more physically flexible designs. For example, NeuroGrid is a flexible organic material-based interface array,^9^ whereas this flexible electrode film primarily allows for the detection of superficial cortical neuron signals.^9^ To facilitate deep-brain measurement, an innovative approach has been the development of injectable neural mesh. This mesh-like structure can be injected into a specific brain region by using a syringe, but the position can no longer be altered postinjection.^11,12^

**Figure 1:**
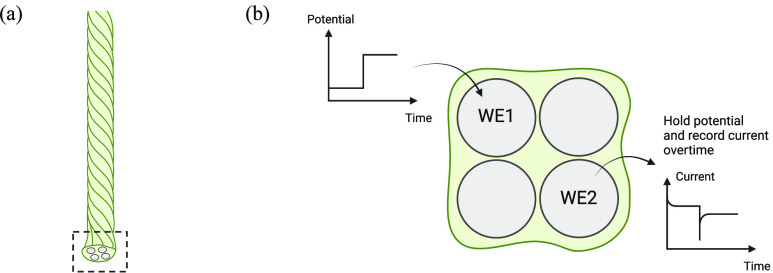
(a) Schematic tetrode. The green color indicates the outer insulation layer. The metal, depicted in gray, is only exposed at the tip. The boxed section is enlarged and shown in (b). (b) Setup to mimic neural recording. A stepped potential is applied to one of the wires on tetrode (WE1), mimicking neural activity. The response experienced by one other wire (WE2) is monitored over time.

The tetrode, a now well-established method, consists of four insulated microwires twisted together, with metal exposed only at the tip (Figure 1(a)). The diameter of a single microwire is typically around 12 to 25 μm,^20−22^ making the tetrode significantly thinner than a silicon probe, which enables it to reach deep-brain regions with minimal damage. Although a single tetrode has only four recording sites, a specially designed drive can hold multiple tetrodes arranged in a custom configuration.^23,24^ This allows for recordings across widely distributed structures, and the drive facilitates vertical adjustments of each tetrode, both before and during recordings.^24^ Advances in drive design now permit compatibility with complex animal movements^20,21^ as well as wireless data logging,^23^ enabling observations of natural and free behaviors. Nevertheless, tetrodes still face an immune response by the brain due to their mechanical mismatch. The mechanical mismatch between the tetrode’s body and the brain tissue can be mitigated by using more bendable materials, such as platinum–iridium instead of tungsten wires.^22^ However, at the tetrode tip, the interface between the rigid electrode metal and the soft brain tissue remains biologically incompatible, limiting the ability to monitor neural activity over extended periods.^1,19^ To maintain the benefits of controllable measurement, one solution is to coat the tetrode interface with a material that is soft, biocompatible, and conductive.

Poly(3,4-ethylenedioxythiophene) (PEDOT) is a conductive polymer that has been used in the coating of implantable neural devices.^25^ PEDOT coatings have been demonstrated to possess good conductivity,^26,27^ low cytotoxicity,^28^ and, importantly, a low level of immune response following in vivo electrode implantation.^29^ Methods for applying PEDOT coatings include spin coating^30^ and electropolymerization.^31,32^ For microelectrodes with densely packed detection sites, the spin coating method may cause inadvertent cross-connections between sites, causing issues for spike sorting and assignment. As a result, electropolymerization is often preferred because it allows for more precise deposition on individual sites. Furthermore, the conditions for polymer film fabrication (e.g., deposition current or potential, duration, and types of dopants, etc.) can be finely tuned to control the process.

**Scheme 1:**
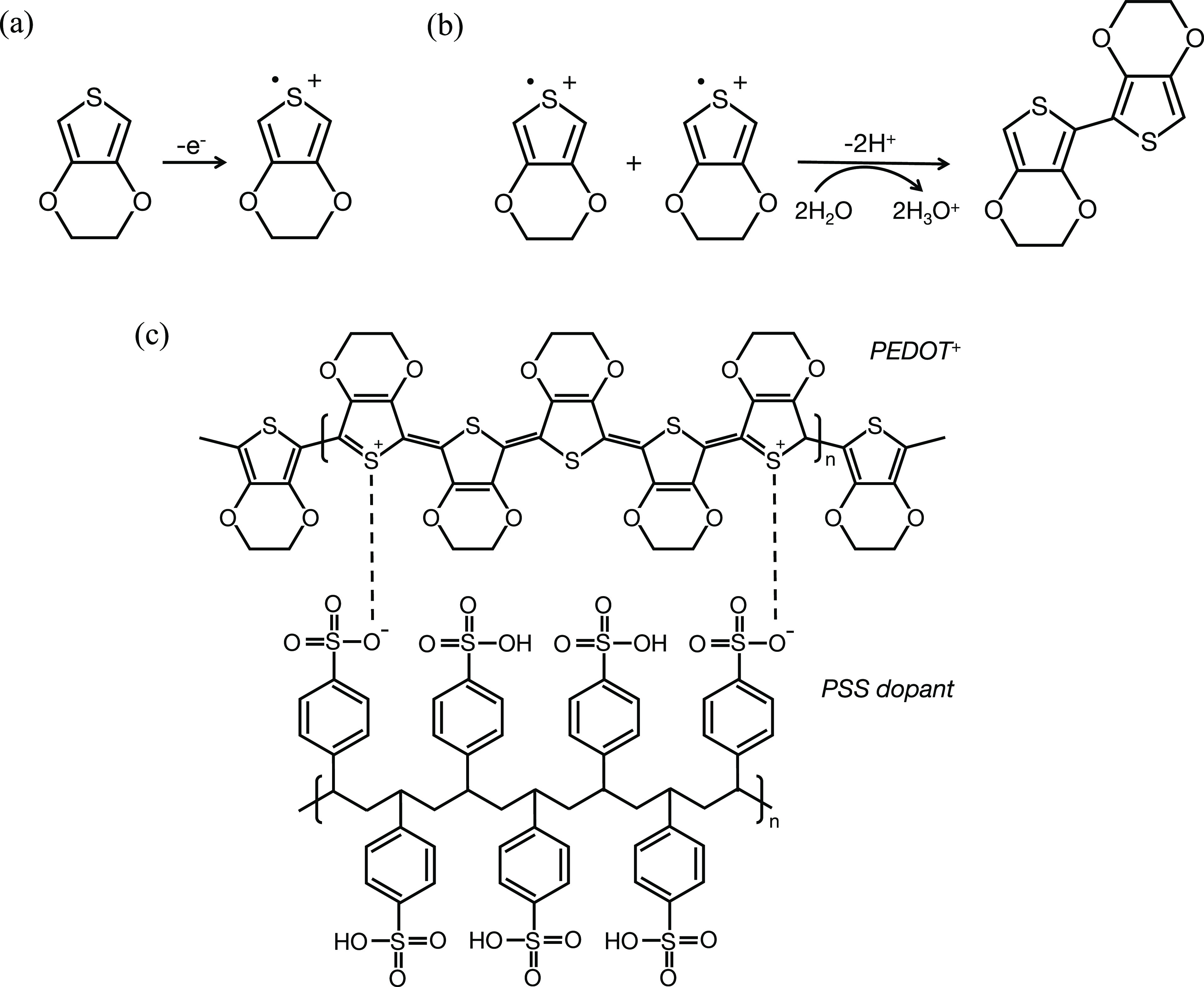
Formation of PEDOT:PSS through Electropolymerization on the Working Electrode Involves a Sequence of Reactions The polymerization process initiates with (a) the oxidation of monomers at the working electrode, followed by (b) the coupling of two oxidized monomers to form a dimer. These dimers are further oxidized to produce oligomeric radicals, which combine with each other and ultimately yield (c) a polymer chain. This chain has a positively charged backbone, requiring the incorporation of PSS^–^ ions or other anions from the solution to neutralize the charge. PEDOT: Poly(3,4-ethylene dioxythiophene); PSS: polystyrene sulfonate.^41^.

An electropolymerization reaction scheme for PEDOT is shown in Scheme 1. The reaction initially involves EDOT monomers in the solution, which form radical cations upon the application of an oxidizing current or potential at the working electrode. As the reaction proceeds, these radicals first couple to form a dimer and subsequently a tetramer. With continued electrochemical oxidation, additional monomeric units join the growing oligomeric chain, leading to the formation of the polymer film. The resultant polymer chain, PEDOT^+^, carries positive charges along its backbone. These charges are neutralized by anions from the solution, which are uptaken so as to stabilize the structure. Various anions have been explored for doping PEDOT^+^, including perchlorate (ClO_4_^–^), benzenesulfonate (BS), p-toluenesulfonate (pTS), tetrafluoroborate (BF_4_^–^), polystyrene sulfonate (PSS^–^), and chloride ions (Cl^–^).^33,34^ The most common material is PEDOT:PSS, which is favored due to its straightforward synthesis,^35^ which is environmentally friendly, not using organic solvents.^35^ PEDOT^+^ can undergo further oxidation at higher potentials, but such overoxidation causes irreversible chemical changes and compromises the electrical conductivity of the polymer film.^36,37^

PEDOT-modified electrodes not only demonstrate improved biocompatibility but also benefit from having lower impedance compared to uncoated electrodes. Previous studies have noted a trend of decreasing impedance with an increase in film thickness, especially at low frequency (<100 Hz).^27,30,38^ However, several researchers have also reported that a thicker coating suffers from delamination, cracking, and reduced biocompatibility.^27,28,39,40^ For stimulating electrodes, thicker films were pursued to improve charge injection capacity to ensure sufficient charge to trigger neural membrane depolarization. Conversely, a massive film is unnecessary for a recording electrode, in particular where little outperformance can be gained over the impedance. Therefore, thickness control is a crucial part of a recording neuron electrode, which has been extensively discussed in our previous paper.^41^

Despite the benefits of PEDOT as a biocompatible conductive polymer, its application has been primarily limited to single, needle-like electrodes^31^ or large, flat slides^30^ rather than to densely packed electrode configurations such as tetrodes. This limitation is partly due to the challenges associated with polymer overgrowth, which can lead to cross-connection of detection sites. These challenges can be overcome through thickness control and optimization. However, a direct method for detecting cross-connection is also essential. Furthermore, while PEDOT-coated electrodes have seen widespread application, the underlying mechanisms of signal recording at the polymer–metal interface remain unclear. Furthermore, the nature of the electrical response within the polymer film created by neural signals is not fully understood. In this study, the electrodeposition of PEDOT is further explored on tetrodes to address these two issues.

Building upon our previous work on thickness control,^41^ this paper presents methods for coating tetrodes with PEDOT:PSS and PEDOT:Cl and systematically characterizes the modified microelectrodes using cyclic voltammetry (CV). Moreover, we propose, for the first time, a simple electrochemical approach to test cross-connection using PEDOT overoxidation, with results supported by optical and SEM images. The details of coating condition selection and polymer characterization are included in the Supporting Information Sections 1 and 2.

In addition, and most importantly, in vitro experiments employing a bipotentiostat to independently control the potentials of two electrodes within a tetrode are reported. During extracellular recording, signals arise due to the depolarization of neuron membranes, which causes local ion flux.^13^ To understand the ion movement during neuronal activity and its contribution to the signals recorded at the tetrode, the potential of one electrode (WE1) within the tetrode can be suddenly altered to replicate the rapid depolarization changes of active neurons (Figure 1(b)). Meanwhile, the response of a nearby second electrode (WE2) within the same tetrode is monitored (Figure 1(b)). In this way, the complex ionic conduction environment in the brain can be simplified, but the essence of signal recording is preserved and can be investigated. In particular, the time scale and magnitude of the response of the monitoring electrode, WE2, is related to the potential change on WE1 and the sensitivity of the monitoring electrode can be compared and contrasted for different surface modifications. Thus, the in vitro experiments that explore how an electrode reacts to adjacent potential disturbances can serve as a basis for understanding signal recording in vivo.

More generally, the procedures for thickness control, electrochemical characterization, and cross-connection assessment are universally applicable to all microelectrodes, particularly those with multiple and densely packed detection sites. The in vitro experiments offer insight into the signal-recording process at the metal–polymer interface, which is the basis for the design and functionality of future neural recording devices.

## 2 Materials and Methods

### 2.1 Materials

Deionized water with a resistivity of 18.2 MΩ·cm at 298 K (Millipore, Millipak Express 20, Watford, UK) was utilized for preparing all solutions. 3,4-Ethylenedioxythiophene (EDOT, 97%), poly(sodium 4-styrenesulfonate) (NaPSS, average Mw = 70 000), phosphate-buffered saline (PBS), and hexaammineruthenium(III) chloride (98%) were purchased from Sigma-Aldrich. Potassium chloride (KCl, 99%) was procured from ThermoFisher Scientific, while sodium chloride (NaCl, >99.5%) was obtained from Scientific Laboratory Supplies.

Pt microwires were supplied by GoodFellow (Pt purity 99.99%, conductor diameter 25 or 15 μm, with polyimide insulation of thickness 5 or 2 μm). Two sizes of Pt microwire were purchased and studied to compare the effects of differing radii. Comparison with the traditional tetrode material, tungsten, was also performed (W purity 99.95%, diameter 12.7 μm coated with Heavy Formvar, obtained from California Fine Wire Company). Details of the electrochemical characterization for 15 μm Pt and 12.7 μm W are included in the Supporting Information Sections 2.1 and 2.2. It should be noted that the microwires discussed in this paper are all insulated wires with only the tip exposing the metallic substrate to the solution, thus functioning like a microwire disk electrode. Unless otherwise specified, the 25 μm Pt microwire was utilized either as a single microwire disk electrode or in constructing tetrodes. Comprehensive procedures for microwire device fabrication and tetrode assembly for electrochemical measurements are provided in the Supporting Information Section 2.

### 2.2 Electrochemical Apparatus and Methods

Electrochemical experiments were carried out using a μ-AutolabIII potentiostat/galvanostat (Autolab B.V., Utrecht, The Netherlands) controlled by NOVA software. The experiments utilized a standard three-electrode setup within a thermostated Faraday cage. The reference and counter electrodes were an SCE (saturated calomel reference electrode, BSi Inc., West Lafayette, IN, USA) and a graphite rod, respectively, for all experiments. All potentials reported in this paper are referenced to the SCE unless otherwise specified (e.g., vs open circuit potential OCP). The cell solution was maintained at 25 ± 1 °C and degassed with nitrogen before each electrochemical experiment. Prior to each use, the Pt microwire or tetrode was cut with fine scissors (Fine Science Tools, 14568–12) to expose a fresh surface(s).

#### 2.2.1 Electropolymerization

The electropolymerization was conducted galvanostatically in a solution of 10 mM EDOT and 0.1 mM NaPSS [0.7% (w/v)]^41^ or 10 mM EDOT and 0.1 M NaCl.^34^ A constant current of 20 nA was applied for 13 s to achieve an average charge deposition density of 50 mC cm^–2^ following our previous protocol.^41^ Additional details regarding the optimization of deposition conditions and polymer film characterization on both micro- and macroelectrodes can be found in the Supporting Information Sections 1 and 2.

#### 2.2.2 Cross-Connection Check

In order to ensure that none of the electrodes within a tetrode were in electrical contact with each other, the coated tetrode was immersed in 0.1 mM NaPSS solution, and a CV scan was performed for each wire from an open circuit potential (OCP) to 1.5 V (vs SCE) and back to OCP, at a scan rate of 50 mV s^–1^. The scan was initiated from OCP so as to avoid solvent decomposition or overoxidation of the polymer. If there is no cross-connection, an overoxidation peak should be visible during the first scan of each wire at a potential around 1.2 V. Conversely, if overgrowth of the polymer has resulted in cross-connection between electrodes, scanning one electrode will also affect the polymer on any cross-connected electrodes, and as a result, no overoxidation peak will be observed during the initial scans of those that are cross-connected. (Details are provided in the Supporting Information Section 2.3)

#### 2.2.3 Bare Pt Characterization

To identify a potential range for Pt in 0.01 M PBS in which undesired Faradaic activity was present, a bare Pt microwire was immersed in 0.01 M PBS, and a CV scan was performed. The scan range was from OCP to various potentials (0.2–1.0 V vs SCE) and then to −0.2 V (vs SCE), returning to OCP at the end with a scan rate of 50 mV/s.

#### 2.2.4 Coated Pt Characterization

For characterization of the coated Pt in 0.01 M PBS and for identifying ranges of potential in which no undesired Faradaic processes took place, a PEDOT:PSS- or PEDOT:Cl-coated Pt microwire was immersed in 0.01 M PBS. A CV scan was executed from OCP to different upper limits (0.5/1.0/1.5 V vs SCE) and then to −0.2 V (vs SCE), and back to OCP at a scan rate of 50 mV/s.

### 2.3 Potential Step Experiments with a Bipotentiostat

**Figure 2:**
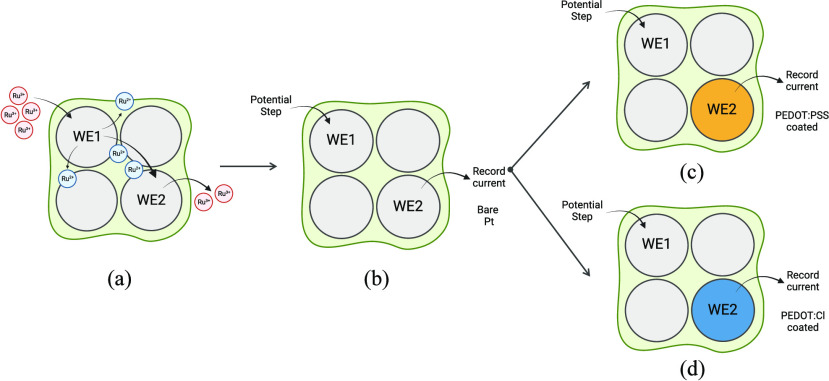
Experiment arrangement. (a) Setup to measure the collection efficiency. Initially, only Ru^3+^ is in the solution, and both electrodes are held at a potential corresponding to the transport limited formation of Ru^3+^ so that no current flows. When the potential at WE1 is swept to the reduction potential, Ru^2+^ is gradually produced, some of which diffuses toward WE2 where Ru^2+^ is oxidized. In this way, WE2 “collects” some of the Ru^2+^ formed at WE1, leading to the current recorded at WE2. (b) Potential step experiment with a bare Pt electrode. A potential step is applied to WE1, and the response is simultaneously recorded at WE2. (c,d) The potential step experiment setup with WE2 coated with (c) PEDOT:PSS and (d) PEDOT:Cl.

Experiments were carried out using an Autolab PGSTAT30 (Autolab B.V., Utrecht, The Netherlands). A four-electrode setup was applied. Two wires of the tetrode (WE1 and WE2) served as the two working electrodes. An SCE was used as the reference electrode, and a graphite rod functioned as the counter electrode. The experimental procedure is illustrated in Figure 2.

#### 2.3.1 Collection Efficiency Measurements Using Hexaammineruthenium(III) Chloride

A bare Pt tetrode was immersed in a 1 mM hexaammineruthenium(III) chloride solution with 0.1 M KCl as a supporting electrolyte. A CV scan on two selected wires was made from 0.2 V (vs SCE) to −0.5 V (vs SCE) with a potential reversal back to 0.2 V (vs SCE) at a scan rate of 25 mVs^–1^ to obtain near steady-state currents for Ru^2+^ oxidation (E1) and Ru^3+^ reduction (E2). The surface of the tetrode was refreshed by cutting before continuing. On WE1, a linear sweep was conducted from E1 to E2 at a scan rate of 5 mVs^–1^, while WE2 was held at the potential of E1. The following reactions happen at each electrode:
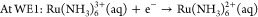
1
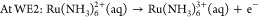
2The process is illustrated in Figure 2(a). Current was recorded at both WE1 and WE2 to calculate the collection efficiency (*N*) using eq 3.^42,43^ The collection efficiency (*N*) measures the ratio of Faradaic current at a detector electrode (WE2) to that at the generator electrode (WE1).^42,43^ In this way, the fraction of the Ru^2+^ species generated at WE1 that have been transported to the “detector” electrode WE2 is quantified:
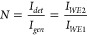
3

#### 2.3.2 Potential Step Experiments with a Tetrode

Potential step experiments were conducted using two electrodes within a tetrode, one of these with either a bare Pt (Figure 2(b)) or a polymer-coated electrode (Figure 2(c,d)) acting as a detector electrode (WE2), with the other electrode (WE1) used to generate signals to mimic neural action. The bare or partially coated tetrode was immersed in 0.01 M PBS. WE2 was maintained at potentials of 0.15, 0.25, or 0.35 V (vs SCE), while a stepped potential was applied to WE1 starting from 0.25 V with a jump to potentials in the range −0.5∼1.0 V (vs SCE). Each step typically was 10 s in duration. Some recorded currents, notably those from polymer-modified surfaces, exhibited fluctuations. In these cases, the data were smoothed using MATLAB (Supporting Information Sections 3.2–3.3).

### 2.4 In Vivo Experiments

One housed adult mouse was implanted with a single microdrive containing 14 independently movable tetrodes. To allow recovery from the surgery, the recording started by the end of the fifth week after the implantation. The surgery and recording procedures reflect previous studies,^20,44^ and the details are included in the Supporting Information section 4.

## 3 Results and Discussion

In this section, two wires of a tetrode are utilized to simulate the signal-recording process and to understand the interactions at the interface. These wires are designated as WE1 and WE2, with WE1 functioning as a signal generator and WE2 serving as a signal recorder. Initially, a model system is used to quantify the diffusion of material generated on WE1 via reduction and then collected and oxidized on WE2. The ratio of the currents is known as the collection efficiency, *N* (0 < *N* < 1). Upon confirming that diffusion to an adjacent electrode is significant (*N* > 0), a series of potential steps is applied to WE1, and the response on WE2 is monitored in 0.01 M PBS. This procedure is conducted successively with WE2, using bare Pt, PEDOT:PSS-coated Pt, and PEDOT:Cl-coated Pt, to compare and elucidate the ion movements responsible for the signal generation.

### 3.1 Collection Efficiency Measurements

**Figure 3:**
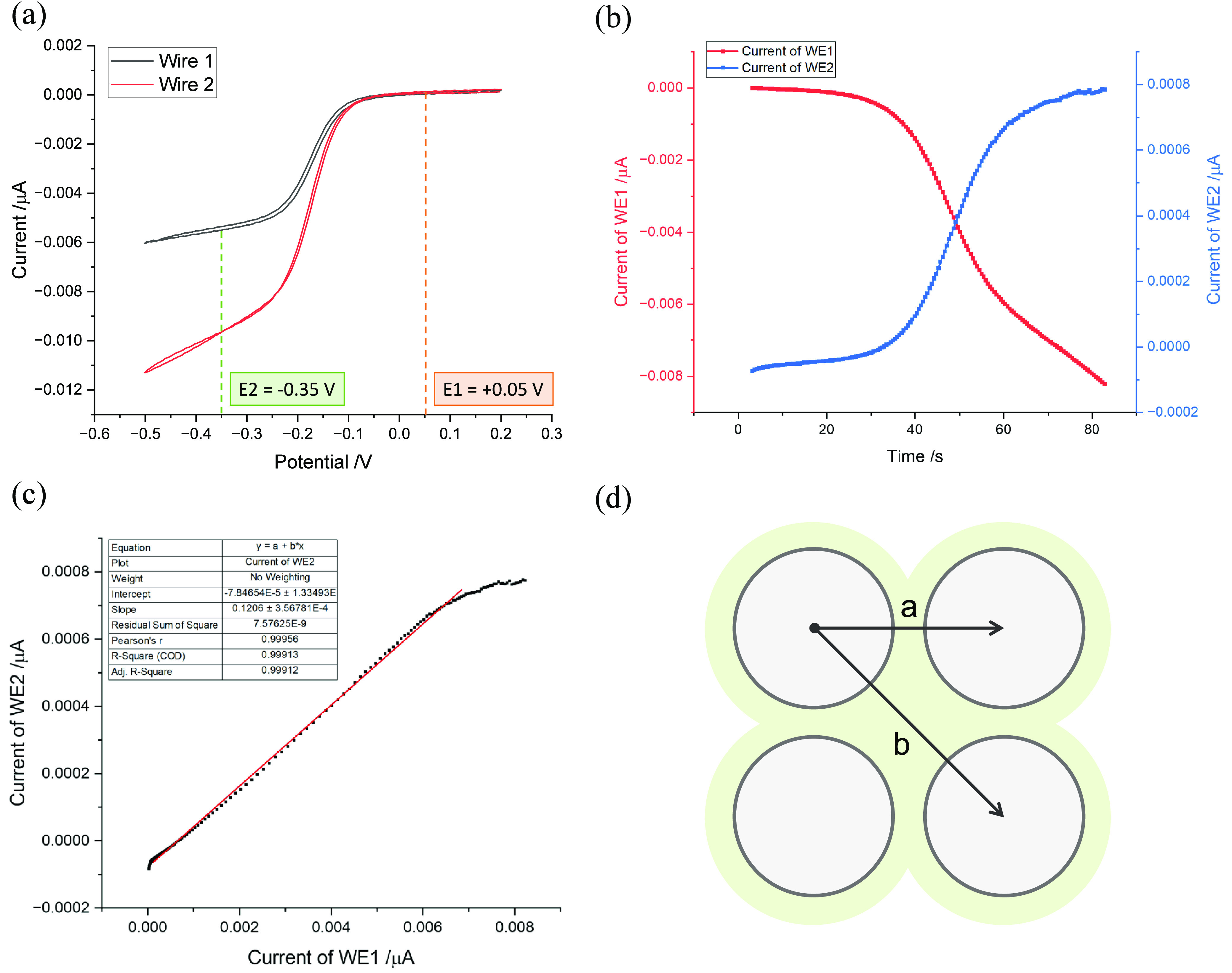
(a) CV scan of Pt tetrode wires from 0.2 → −0.5 → 0.2 V in 0.1 mM hexaammineruthenium(III) chloride solution with 0.1 M KCl as a supporting electrolyte (scan rate, *v* = 25 mVs^–1^). (b) The current recorded on WE1 and WE2 when a slow linear sweep (*v* = 25 mVs^–1^) of potential on WE1 is made from an oxidation potential (0.5 V) to a reducing potential (−0.35 V) while holding the potential on WE2 constant at an oxidizing potential of 0.5 V. (c) The plot of current on WE2 against WE1 is fitted with a linear line (red line), and the gradient is used to obtain the collection efficiency. (d) Graphic illustration of the tetrode cross-section. “a” denotes the length of the adjacent discs, and “b” denotes the diagonal distance between discs. All potentials are reported relative to the SCE.

A bare Pt tetrode was immersed in a solution with 1 mM hexaammineruthenium(III) chloride and 0.1 M KCl, and an initial potential scan was performed on two selected wires, starting from 0.2 V and scanning cathodically to −0.5 V before returning to 0.2 V (vs SCE). The resulting CV scans, as illustrated in Figure 3(a), show near zero current regions at more positive potentials and transport controlled regions at sufficiently negative potentials; a half wave potential for the Ru^2+^/ Ru^3+^ redox couple was estimated (ca. −0.15 V vs SCE), which is in good agreement with literature reports.^45^

Subsequently, the tetrode was freshly cut to expose clean Pt to the solution. Both wires were connected to the bipotentiostat, with WE2 held at a potential (E1) of 0.05 V (vs SCE) throughout the experiment, while WE1 underwent a linear potential scan from E1 to E2 (−0.35 V vs SCE) corresponding to transport-controlled reduction of Ru^3+^. The resulting currents on WE1 (red line) and WE2 (blue line) are presented in Figure 3(b). Initially, the current signals at WE1 and WE2 were near zero because the solution contained only Ru^3+^, and both electrodes were at the oxidation potential, precluding any reaction. However, as the potential on WE1 was swept toward E2, Ru^2+^ began to form at WE1 (eq 1), as indicated by the increasingly negative current (red line). WE2, in turn, captured the diffused Ru^2+^, triggering oxidation (eq 2) and leading to an influx of Faradaic current, as evidenced by the rising potential (blue line) toward more positive values.

By comparing the current recorded on WE1 and WE2, the efficiency of the Ru^2+^ collected on WE2 from WE1 can be calculated using eq 3, which graphically is the gradient of Figure 3(c). The ratio of the currents is known as the collection efficiency, *N*, where 0 < *N* < 1 with the fraction 1 – *N* reflecting material generated at WE1, which is lost to the bulk solution. Figure 3(c) shows that the collection efficiency *N* = 0.12 ± 0.04, which relates to the specific electrodes studied as the electrode size can vary within the array. To estimate the distance between electrode discs, as depicted in Figure 3(d), with each wire having a radius (*R*) of 12.5 μm and an insulation thickness (*t*) of 5 μm, the adjacent (*a*) and diagonal (*b*) distances to neighboring electrodes was calculated as the following:
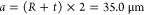
4
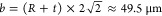
5The findings show that changes on one tetrode disc can affect an adjacent disc, with around 10% of the ions diffusing to neighboring electrodes. Additionally, the distances between discs, whether adjacent or diagonal, are within the range for extracellular recording (< ∼140 μm^1,14^). This encourages the use of potential step experiments using the tetrode to mimic neuronal recording as described in the next section.

### 3.2 Potential Step Experiments on a Bare Tetrode

In this section, we explore how the current response on a single electrode within a tetrode array responds to a potential step on a nearby electrode, providing a basis for understanding the responses induced by neural potential transients.

**Scheme 2:**
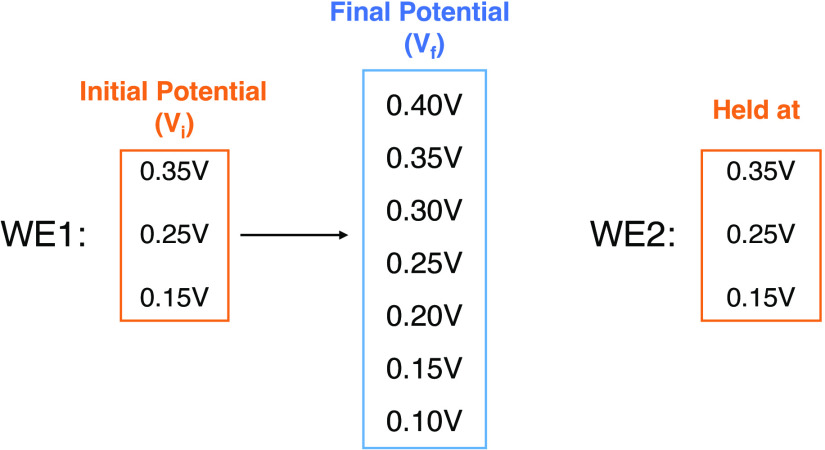
Potential Step Experiment Using Bare Pt in 0.01 M PBS A series of potential steps are applied to the working electrode 1 (WE1) with all potentials within the range of the region without Faradaic activity. The potential of the working electrode 2 (WE2) is fixed at the starting potential of WE1. All potentials are reported relative to the SCE.

**Figure 4:**
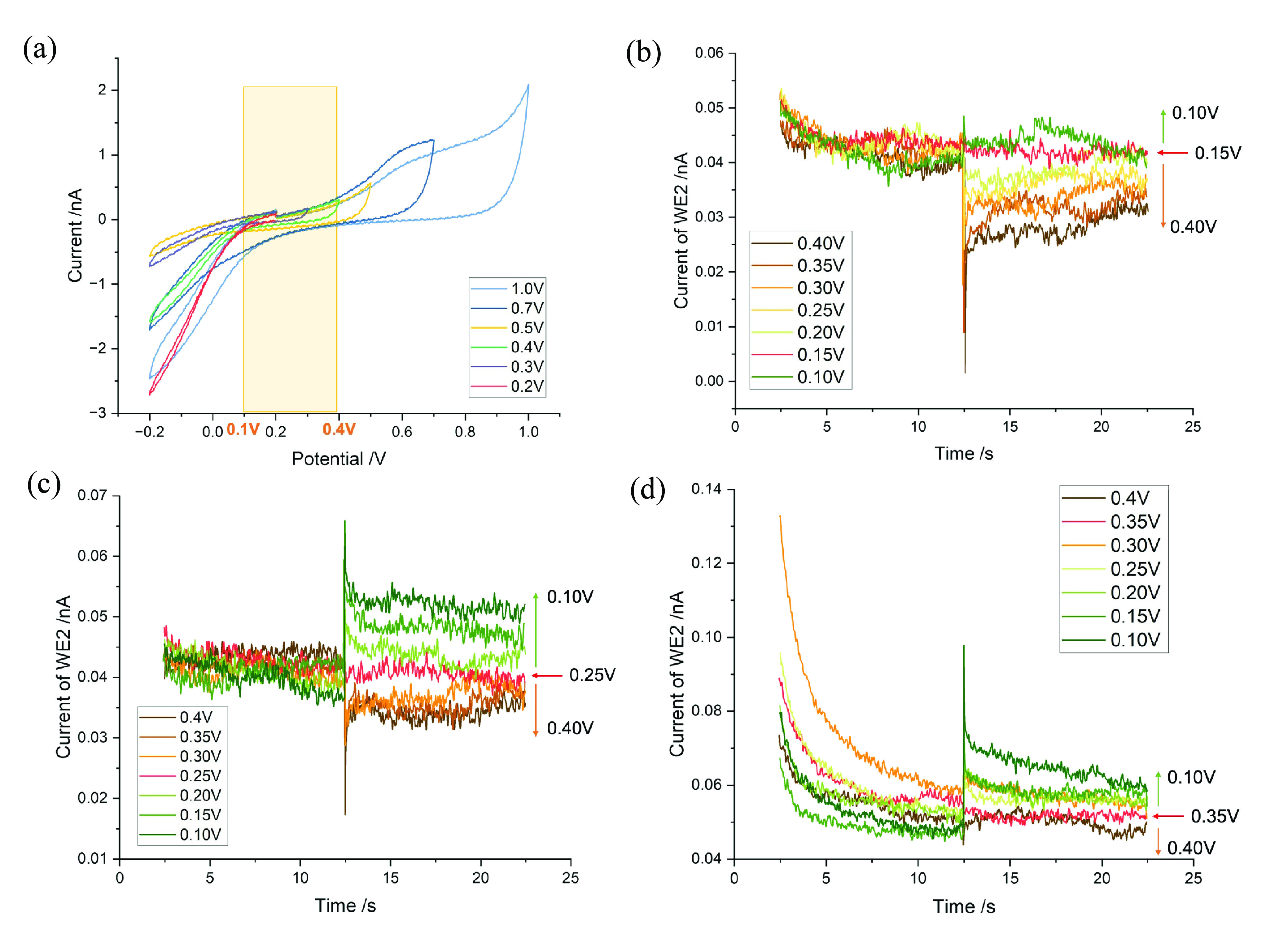
(a) CV scan of a bare Pt microwire in 0.01 M PBS from OCP → 0.2–1.0 → −0.2 → 0.2 V. The region without Faradaic activity is highlighted in yellow (0.1∼0.4 V) (scan rate, *v* = 50 mVs^–1^). (b–d) Current detected on WE2 when a potential step was applied to WE1 (0.15 V/0.25 V/0.35 V → 0.10–0.40 V), holding WE2 at 0.15 V/0.25 V/0.35 V respectively. The red arrow represents the potential at which WE2 was held. The green arrow indicates the direction of the final potential is more negative than the WE2 potential, while the orange arrow indicates the opposite. All potentials are reported relative to the SCE.

Before initiating the potential step experiments, it is necessary to identify a potential range that avoids undesired (electro-)chemical reactions that would result in unwanted Faradaic currents, for example, due to solvent decomposition or surface oxide formation. A CV scan was conducted on a bare Pt microwire immersed in 0.01 M PBS, setting the potential window from a fixed minimum of −0.2 V to a range of maximum potentials between 0.2 and 1.0 V (vs SCE) with the aim of locating a region for Pt in PBS in which Faradaic activity is absent and only capacitive charging observed. As indicated by the yellow box in Figure 4(a), a potential range from 0.1 to 0.4 V (vs SCE) was identified in which no current peaks or shoulders were observed, suggesting the absence of electrochemical reactions. Consequently, the initial potential for the stepped potential on WE1 and the holding potential on WE2 were selected within this range. Starting potentials (*V_i_*) of 0.15, 0.25, and 0.35 V were chosen to be evenly distributed within the unreactive region. The final potential (*V_f_*) was set to span the entire range from 0.1 to 0.4 V. The experimental procedure is detailed in Scheme 2. The currents recorded on WE2 are presented in Figure 4(b–d). Observations from Figure 4(b–d) reveal a consistent pattern: if the final potential is more positive than the initial potential (*V_f_* > *V_i_*, orange arrow), the current on WE2 exhibits a rapid negative pulse before gradually returning to a steady-state current. Conversely, if the final potential is more negative than the initial potential (*V_f_* < *V_i_*, green arrow), the current on WE2 shows a rapid positive pulse, also decaying to a steady-state level over time.

**Figure 5:**
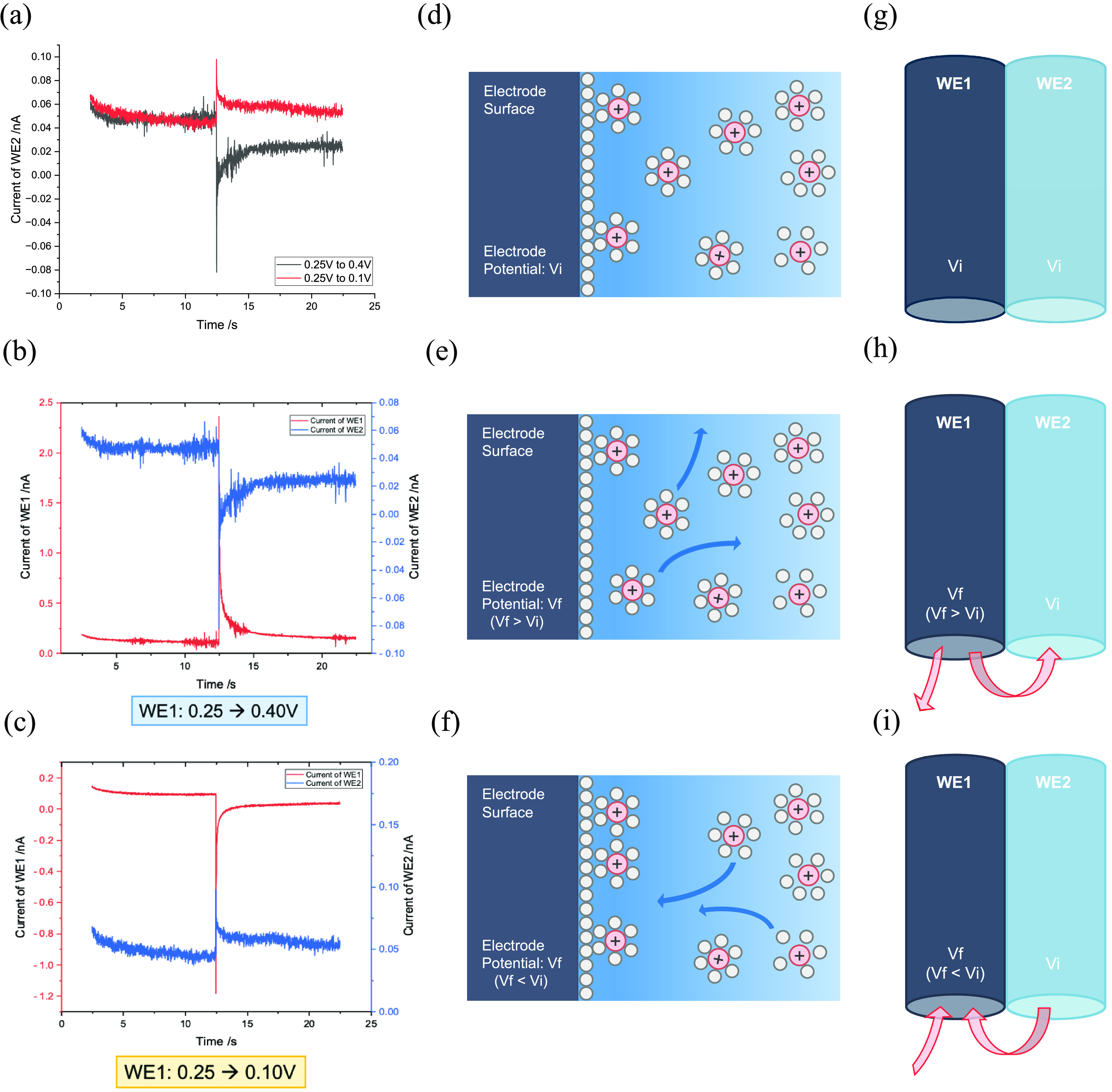
Left column: (a) Current transients recorded on WE2 for potential steps on WE1 from 0.25 → 0.1 and 0.4 V. (b,c) Current transients on WE1 (red line) and WE2 (blue line) for WE1 stepped (b) from 0.25 to 0.4 V and (c) from 0.25 to 0.1 V. All potentials are reported relative to the SCE. Middle column: Schematic showing inferred ion motion on the WE1 surface. (d) Initial double layer, (e,f) Ion movement when the final potential is more positive or more negative than the initial potential; positive charges are repelled away from or attracted to the surface, creating a positive or negative current pulse on WE1. Right column: Coupled response between WE1 and WE2. The pink arrow indicates the direction of cation flow. (g) Initially, both electrodes have a double layer reflecting the potential of the electrode. (h,i) Early in the transients, the charges near WE1 are repelled/attracted over a short time scale, so that ion movement near WE2 is the inverse to that of WE1, producing an opposite direction of the current pulse. *V_f_* denotes the final stepped potential, and *V_i_* represents the initial potential.

To better understand the process, the current responses on electrodes for potential steps from 0.25 to 0.1 and 0.4 V were studied in further detail (Figure 5). Figure 5(a) displays the current only on WE2, where distinct positive and negative current pulses are evident for different final potentials. Then, to examine the current response on both electrodes, the currents on WE1 (red line) and WE2 (blue line) are overlaid for each case of the pulse (0.25 → 0.1 V/0.4 V) in Figure 5(b,c). Noting the absence of Faradaic activity, the currents must reflect the attraction and/or repulsion of ions at the electrodes, the nature of which can be inferred from the chemical composition of the solution and the direction of current flow. The ionic composition of PBS is detailed in Table 2, alongside a comparison with extracellular fluid (ECF) and artificial cerebrospinal fluid (ACSF). Schematic illustrations of the inferred ionic movement near the electrode surface are presented in Figure 5(d–i).

**Table 2:** Ionic Composition of 0.01 M PBS, Extracellular Fluid (ECF), and Artificial Cerebrospinal Fluid (ACSF)^a^

Ions	0.01 M PBS/mM	ECF/mM^46^	ACSF/mM
Na^+^	138	147	150
Cl^–^	140	113	155
K^+^	2.70	2.90	3.0
	10.0	0.358	1.0
Ca^2+^	/	1.14	1.4
Mg^2+^	/	1.10	0.8
	/	23.3	26

a The ionic compositions of PBS and ACSF (catalog number: 352525 ML, Fisher Scientific) are obtained from the product information from their supplier.

Initially (*t* < 10 s), before the change of potential, both working electrodes attain a near steady-state current close to zero following the formation of the double layer and any other surface processes (Figure 5(d,g)). When the final potential (*V_f_*) is more positive than the initial (*V_i_*) (Figure 5(e)), the positive charge near the WE1 surface is repelled once the potential step occurs (Figure 5(e)), producing a large and sudden positive increase in the current on WE1 (Figure 5(b), red line). Concurrently, this pulse also disrupts the ion distribution at the adjacent WE2, leading to an influx of positive charges and a decrease in detected current, which corresponds to the outward flow of positive charge from the electrode (Figure 5(b), blue line; Figure 5(h)). Eventually, the current reaches a steady value due to the completion of a new ion distribution around the electrode. After sufficient time, a small steady-state background current on both electrodes flows, giving the long and flat tail at the end. Conversely, when *V_f_* is more negative than *V_i_* (Figure 5(c)), a rapid influx of positive charge toward WE1 occurs (Figure 5(f)), resulting in a negative spike in current (Figure 5(c), red line). Simultaneously, positive ions near WE2 are drawn toward WE1, increasing the positive charge outflow at WE2, and thus, a larger current is observed (Figure 5(c), blue line; Figure 5(i)).

It is important to note that the transient spikes are attributable to ion movement within the diffusion layer when there is a sudden change in applied potential, while the flat steady-state current at the extremities is the Faradaic current due to trace amounts of electrolysis at the surface.

### 3.3 Potential Step Experiments on PEDOT-Coated Tetrodes

**Scheme 3:**
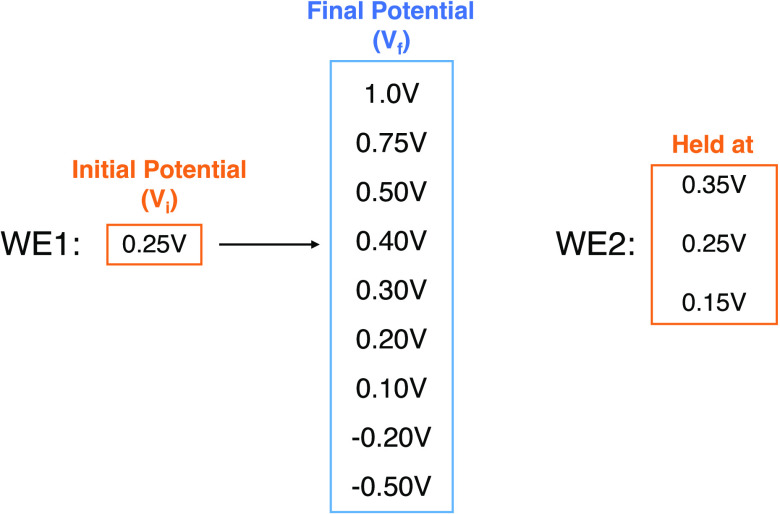
Potential Step Experiment Using a PEDOT-Coated Pt in 0.01 M PBS A potential step was applied to the working electrode 1 (WE1) with a fixed starting point to a range of final potentials. The potential of the working electrode 2 (WE2) was fixed at 0.15, 0.25, or 0.35 V. All potentials are reported relative to the SCE.

Next, potential step experiments were carried out in which WE2 was coated with a PEDOT polymer using different dopants: PSS^–^ or Cl^–^, and the results compared to those of bare Pt. For these experiments, WE1 was set to a fixed initial potential of 0.25 V (vs SCE), selected as this is the potential in the middle of the unreactive range (as discussed above) and close to the OCP of Pt in 0.01 M PBS (ca. 0.2 V vs SCE). A broad range of final potentials, from −0.5 to 1.0 V (vs SCE), was investigated. This potential range was chosen based on the unreactive range identified for PEDOT-coated Pt in PBS (Supporting Information Section 3.1). The potential on WE2 was held constant at 0.15, 0.25, and 0.35 V (vs SCE), as in the previous section. The experimental procedures are outlined in Scheme 3.

#### 3.3.1 PEDOT:PSS-Coated Tetrode

**Figure 6:**
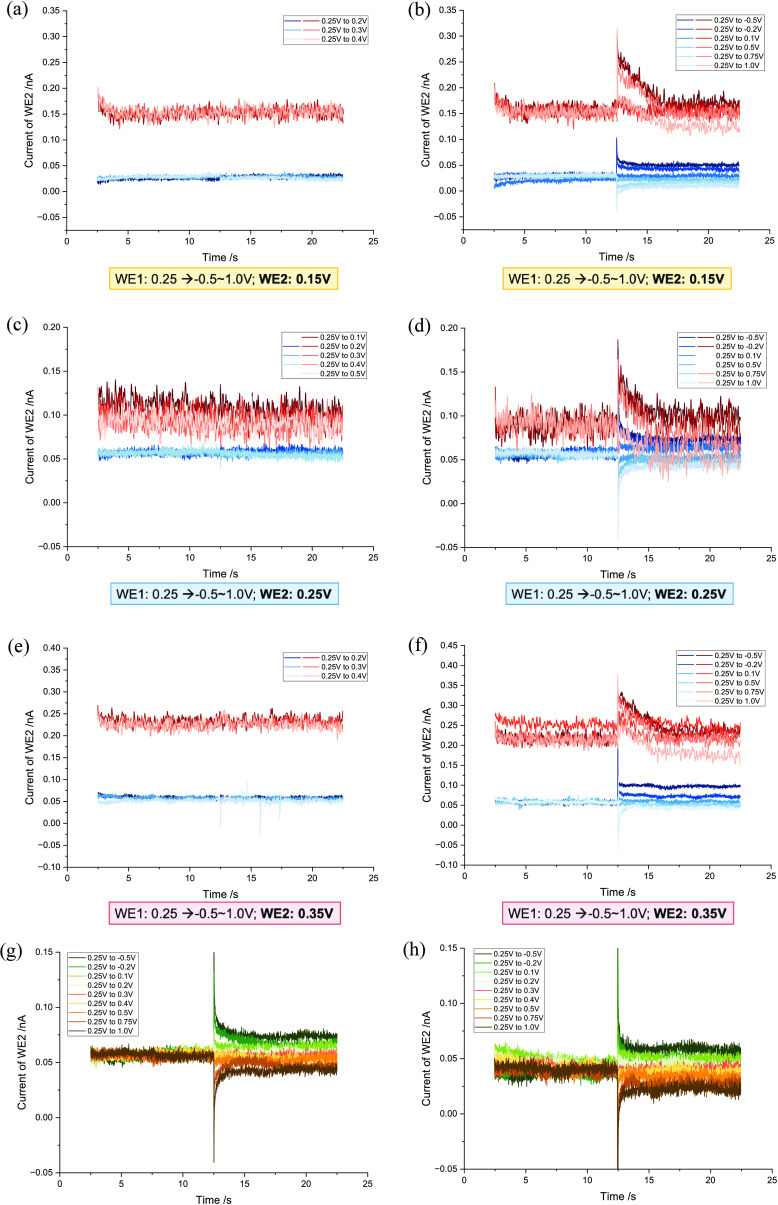
(a–f) The current recorded on WE2, where WE2 was held at 0.25 V/0.15 V/0.35 V, respectively, and the potential on WE1 jumped from 0.25 V to a range of final potentials. The left column graphs (a,c,e) illustrate the situations where the potential step on WE1 triggered little response on WE2. The right column graphs (b,d,f) include the potential steps that led to significant responses on WE2. *Blue lines*: Bare Pt. *Red lines*: PEDOT:PSS-coated Pt. (g,h) Potential step experiment with WE2 held at 0.25 V, and WE1 jumped from 0.25 V to a range of values, using (g) a bare tetrode and (h) an overoxidized PEDOT-coated tetrode. All potentials are reported relative to the SCE.

The PEDOT:PSS coating and characterization is summarized in the Supporting Information Section 2.2.1. Following the same procedure as the bare Pt, the nonreactive potential range for PEDOT:PSS-coated Pt microwire was determined to be from −0.5 to 1.0 V (vs SCE) (Supporting Information, Figure S12(a)). After coating WE2 with PEDOT:PSS, the potential step experiment was carried out, and the procedure was repeated with a bare Pt tetrode for comparison. The recorded current on WE2 after data smoothing is presented in Figure 6.

In Figures 6(a,c,e) (left column), the spikes observed in WE2 are relatively minor or barely noticeable when the end potential on WE1 approximates the starting value of 0.25 V (e.g., *V_f_* = 0.2∼0.4 V). In contrast, Figures 6(b,d,f) (right column) show more pronounced spikes when the potentials on WE1 finished at values significantly different from 0.25 V (e.g., *V_f_* = −0.2, −0.5, 0.75, and 1.0 V). This pattern was consistent across both bare and coated electrodes.

Furthermore, comparing the coated and the bare Pt electrodes, the coated electrodes detected more positive current than the bare ones, in particular when WE2 was held at 0.15 and 0.35 V. The final steady-state currents showed that the more negative the final potential relative to the initial, the more positive the final steady-state current, and vice versa, which is consistent with the observation with bare Pt (Figure 4(b–d)). However, the most significant contrast is that the spikes for PEDOT:PSS-coated Pt consistently showed only positive current values, irrespective of the final potential, in contrast to both the positive and negative currents seen with bare Pt.

To discern which component of the polymer contributes to the unidirectional current flow, PEDOT:PSS-coated electrodes were examined more closely. Note that the polymer comprises PEDOT^+^, facilitating hole transport, and PSS^–^, with the latter more involved in ion transport.^30,35,47^ With the aim to study the origin of the unidirectional current from either PEDOT^+^ or PSS^–^, the PEDOT:PSS was first coated onto WE2, and then a CV scan was applied ranging from OCP to 1.5 V (vs SCE) to overoxidize the PEDOT^+^. The overoxidation step destroyed the PEDOT^+^ electrical conductivity, changing the polymer film charge structure, and the process is known to involve counterion flux (PSS^–^) leaving the film.^48^ The potential step experiment was then conducted with WE2 held at 0.25 V, and the current observed on WE2, as shown in Figure 6(g), exhibited little difference from that of bare Pt (Figure 6(h)), suggesting that the presence of PSS^–^ may account for the unidirectional current flow. Therefore, further research was pursued to evaluate the impact of dopants by doping PEDOT^+^ with another ion, Cl^–^, for comparison.

#### 3.3.2 PEDOT:Cl-Coated Tetrodes

The procedure and results of PEDOT:Cl coating characterization and optimization are detailed in the Supporting Information Section 1 and 2.2.2. Similarly, the nonreactive region for PEDOT:Cl-coated Pt in 0.01 M PBS was determined to be between −0.5 and 1.0 V (vs SCE) (Supporting Information Figure S12(b)). Following the same methodology in Scheme 3, a potential step experiment was performed.

**Figure 7:**
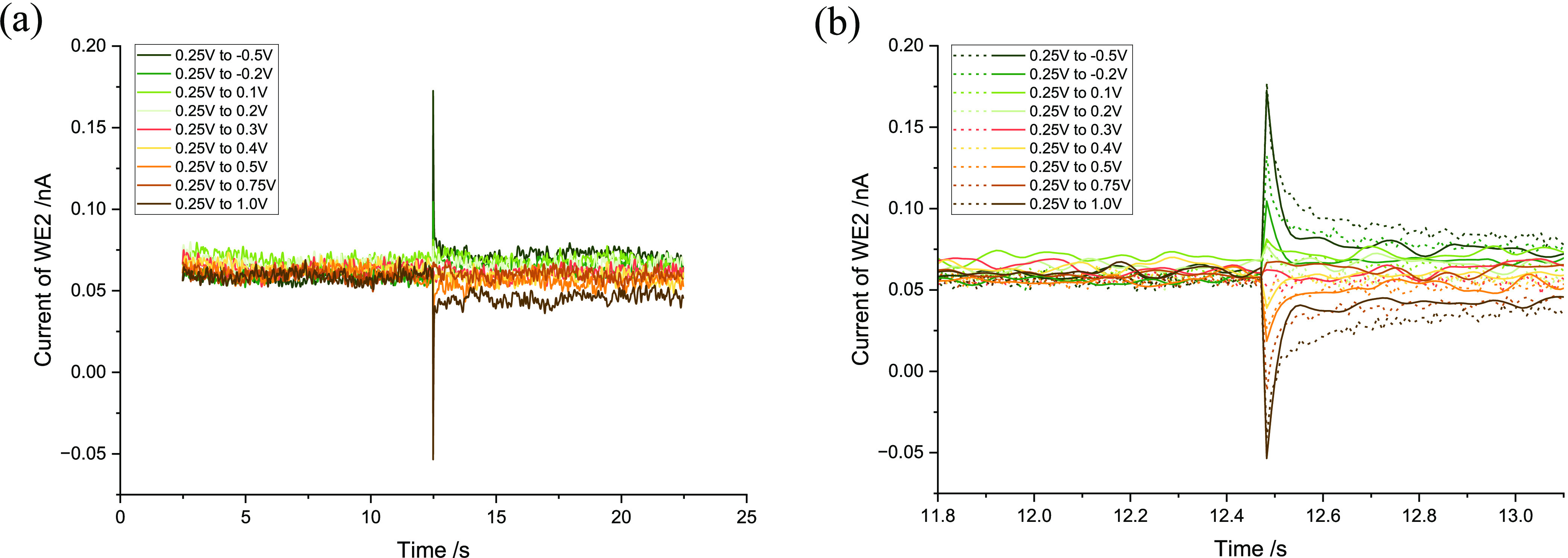
Current recorded on WE2, where WE2 was held at 0.25 V, respectively, and the potential on WE1 jumped from 0.25 V to a range of final potentials. (a) WE2 was coated with PEDOT:Cl. (b) Zoom in and comparison of the current immediately before and after the potential step between the PEDOT:Cl coated and the bare WE2. *Solid line*: PEDOT:Cl-coated. *Dashed line*: Bare Pt. All potentials are reported relative to the SCE.

Similarly to what was observed with PEDOT:PSS, a small potential step (*V_f_* = 0.2∼0.4 V) produced only a minor current pulse, whereas larger potential changes led to more pronounced current pulses. The results are detailed in the Supporting Information Section 3.2.2. After the coating, the final steady-state current values for WE2 at 0.15 and 0.35 V were more positive than those for a bare Pt electrode, while the current with WE2 held at 0.25 V was generally comparable to that of a bare Pt electrode. Most notably, the current flow in both positive and negative directions was restored (Figure 7(a)), with the current direction depending on the initial and final potentials, the same pattern as observed with the bare Pt in Section 3.2. Additionally, both the pulse and subsequent recovery of the double layer occurred at a noticeably faster rate for the PEDOT:Cl-coated electrode compared to the bare Pt. This is evident in Figure 7(b), where the capacitive decay indicated by solid lines (PEDOT:Cl-coated) was quicker than that indicated by dashed lines (bare Pt), and the steady-state current was rapidly restored following the potential step.

On the basis of the above and noting that the bidirectional pulse can be restored either by excluding PSS^–^ through overoxidation or by employing alternative counterions such as Cl^–^, it can be concluded that the unidirectional current flow is associated with the presence of PSS^–^ in the polymer matrix. The insensitivity of PSS^–^ to anion movement is likely due to its large chain structure and strong doping with PEDOT^+^ (as depicted in Scheme 1), which inhibits its mobility in and out of the polymer, thus affecting signal transmission. Conversely, the smaller Cl^–^ ions can move more freely into and out of the film, enhancing signal transmission. Additionally, the presence of Cl^–^ in both the film and the solution could promote signal propagation, potentially leading to a more rapid signal response.

### 3.4 Comparison of Transient Responses at Different Electrodes

**Figure 8:**
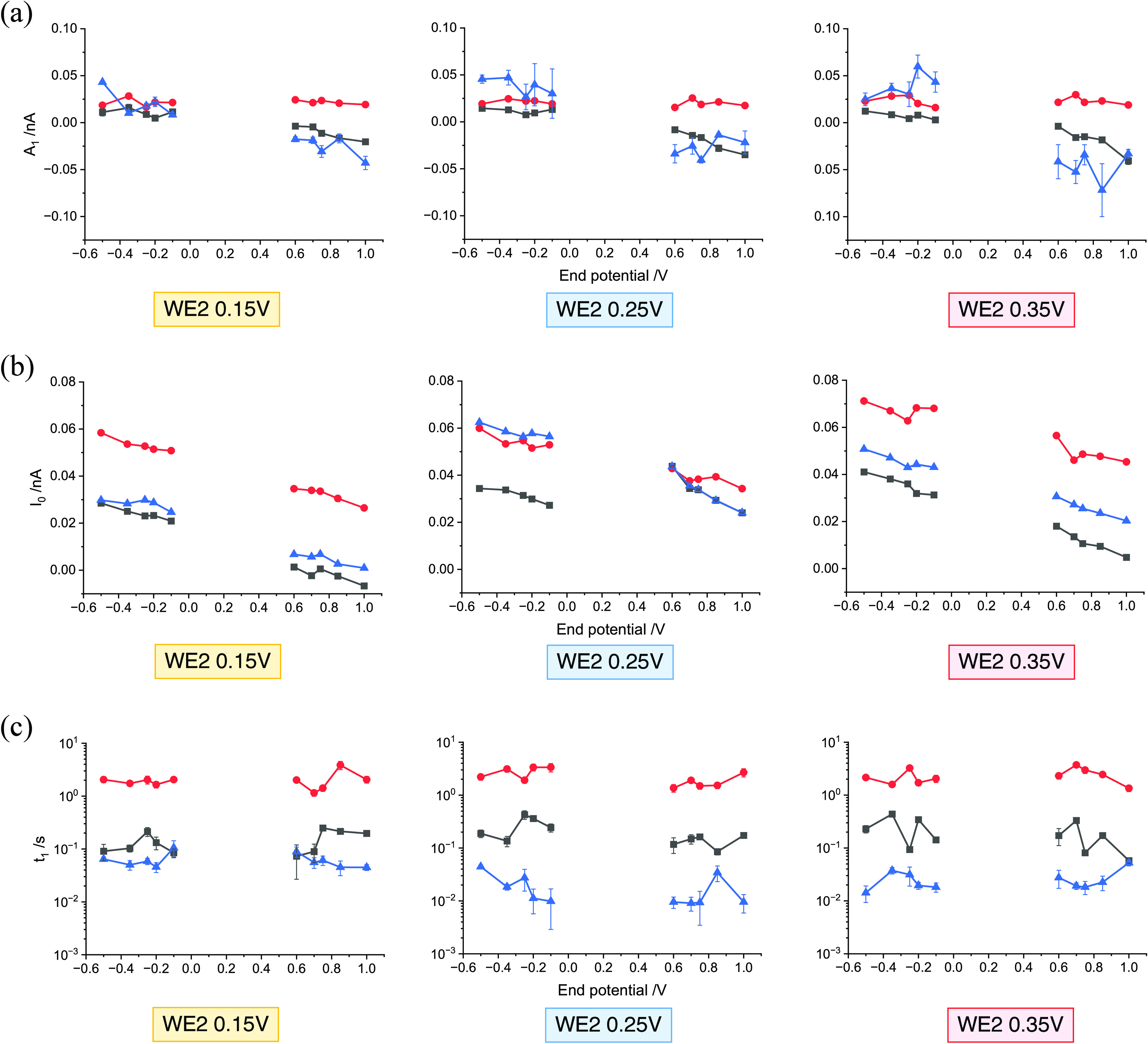
Results of the fitting parameter using eq 6 on the potential step experiments, where WE2 was held at 0.25 V/0.15 V/0.35 V, respectively, and the potential on WE1 jumped from 0.25 V to a range of end potentials (−0.5∼0.1 V and 0.6∼1.0 V). (a) Comparison of the fitting parameter *A*_1_. (b) Comparison of the steady-state current *I*_0_. (c) Comparison of the response time *t*_1_. *Black line*: Bare Pt. *Red line*: PEDOT:PSS-coated. *Blue line*: PEDOT:Cl-coated. All potentials are reported relative to the SCE.

To gain further insights and quantify ion movements at the polymer-solution interface, the current recorded at WE2 after the application of the potential step was approximately fitted with an exponential decay curve using the following equation:
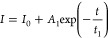
6*I*_0_ represents the steady-state current after the potential step. *t*_1_ is the response time, reflecting how ions around WE2 respond to the sudden potential change at WE1. *A*_1_ indicates the direction and implies the size (*I*_0_ + A_1_) of the resulting current, where a positive *A*_1_ suggests a positive current, and vice versa. It is important to note that the fitting of eq 6 focuses on the transient decay following the initial rapid spike (<0.02 s). Examples of fitting are presented in the Supporting Information Section 3.3. Additionally, to avoid tiny current pulses and so to better fit the exponential curves, large potential steps were chosen to obtain significant responses. Specifically, *V_f_* was chosen to satisfy |*V_f_* – *V_i_*| > 0.35 V, with *V_i_* = 0.25 V (Supporting Information Section 3.3Table 2). The results of the parameters *A*_1_, *I*_0_, and *t*_1_ are summarized in Figure 8.

From Figure 8(a), it can be seen that PEDOT:PSS-coated electrodes consistently produce only positive pulses, and the *A*_1_ values are invariably with a value of 0.022 ± 0.001 s across all transients. In contrast, both PEDOT:Cl-coated and bare Pt electrodes exhibit bidirectional transient decays, their *A*_1_ values hence could be positive or negative depending on the potential step. However, the average magnitude of |*A*_1_| is generally larger for PEDOT:Cl-coated Pt than for bare Pt, with differences of approximately 0.012, 0.016, 0.030 nA, increasing with the WE2 holding potential from 0.15 to 0.35 V. Observing the steady-state current *I*_0_ postpotential step application in Figure 8(b), it is evident that for WE2 = 0.15 and 0.35 V, the values follow the trend PEDOT:PSS > PEDOT:Cl > Bare Pt, reflecting relative amounts of trace Faradaic currents. For WE2 = 0.25 V, the steady-state current is comparable for PEDOT:PSS and PEDOT:Cl, which could be attributed to varying background effects and/or the different morphology of the polymer materials. Lastly, Figure 8(c) compares the response times, *t*_1_, for the transient decay of the three different WE2 electrodes. From the graphs, PEDOT:Cl (blue lines) response is the quickest (average 0.04 ± 0.02s) followed by bare Pt (black lines, average 0.22 ± 0.04 s) and the movement of ions in PEDOT:PSS (red lines) takes the longest time of an average 2.2 ± 0.2 s. Furthermore, the response time was compared with the estimated time scale of pure one-dimensional diffusion between WE1 and WE2:
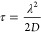
7With an approximate value of *D* = 10^–9^ m^2^ s^–1^, which is typical for ions in aqueous solution and λ = 35–50 μm (using the length of *a* and *b* in Figure 3(d)), the diffusion response time τ is calculated to be between 1.3 and 0.6 s from eq 7. This diffusion response time is on the same order of magnitude as that of bare Pt. The presence of the polymer with different dopants modifies the response time from free diffusion by either 1 order of magnitude less or more, and these differences may arise from the mobility of the anions within the polymer matrices (estimated thickness is of ca. 0.3 μm) and their capacity to enter and exit the polymer. Specifically, the presence of Cl^–^ dopant accelerates the response to approximately within an order of 10^–1^ s, suggesting high ion mobility, while PSS^–^ extends the response to an order of magnitude of around 10 s, indicative of hindered ionic movement. In this case, it is possible that the response time reflects ion motion within pores or channels within the polymer film, restricting the current flow, while in the case of films containing Cl^–^, the response of WE2 is controlled by the exchange of Cl^–^ into and out of the film where they are present at a concentration of ca. 0.1 M in PBS (as shown in Table 2) and estimated from the charge passed in the film growth to be around 18 M in the doped film.

## 4 Biocompatibility Experiments in Vivo

A pilot experiment utilizing a coated tetrode was conducted to assess the biocompatibility of PEDOT-coated Pt electrodes and compared to that of bare Pt electrodes. Details of the procedure are provided in the Supporting Information section 4. The electrode was implanted in the mouse brain for 5 weeks prior to initiating recordings at the hippocampal layers. All polymer-coated electrodes successfully recorded signals, indicating good biocompatibility. Compared to bare Pt in this trial of experiment, the PEDOT-coated electrodes demonstrated enhanced capability to distinguish between neuronal signals. Specifically, the maximum number that the PEDOT:PSS tetrode could distinguish was up to six single-unit recordings, while PEDOT:Cl identified and differentiated seven distinct action potentials from neurons. In contrast, a bare Pt electrode was only able to maximally differentiate spike signals from four neurons.

In addition, to compare the consistency of single-unit recording signals, a comparison factor waveform score (*wv*_*score*_) was introduced:^44^
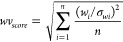
8where *w*_*i*_ is the value of mean waveform of a sample *i*, *σ*_*wi*_ is the standard deviation across all spike of sample *i*, and *n* is the number of waveform samples^44^ (examples of good and bad *wv*_*score*_ waveforms are included in the Supporting Information Figure S18.). The average *wv*_*score*_ of all recorded units was PEDOT:Cl (1.2 ± 0.2) ≈ Pt (1.2 ± 0.1) > PEDOT:PSS (1.1 ± 0.2). Considering PEDOT:Cl and PEDOT:PSS recorded more neuronal signals of different origin, the slight larger error bar accounts for the variety of signals recorded. The results of *wv*_*score*_ for PEDOT:Cl and PEDOT:PSS in comparison with all the *wv*_*score*_ are presented in Supporting Information Figure S19. Compared to PEDOT:PSS, PEDOT:Cl shows better recording quality as the distribution favors the high score sides.

Overall, due to the nature of in vivo experiments, as opposed to in vitro, the neuronal signals generated cannot be identical each time. The recorded waveform largely depends on the proximity and the orientation of the neuron relative to the electrode, and the number of signals recorded can also be significantly influenced by the distribution of neurons. Given the complexities of the extracellular environment, a straightforward comparison is challenging, and further studies are required to elucidate the processes occurring in vivo. However, the results from this pilot study have ensured the biocompatibility of the material and provide a confidence and guidance in further investigation and experimental design.

## 5 Conclusions

New insights into the design of polymer modifications to recording electrodes have emerged from the studies reported. In particular, the potential step results revealed that the choice of PEDOT dopant affects the responses to the signal. For PEDOT:PSS, the strong interaction between the lengthy PEDOT^+^ chains and PSS^–^ restrains the polyanion mobility leading to a slow response time and intrinsic insensitivity to anion flows. Conversely, for PEDOT:Cl, the small Cl^–^ ions, which are present in both the polymer film and the environment solution, diffuse faster, both in solution and inside the film, thus resulting in a quicker response.

In conclusion, the method for tetrode coating and the subsequent cross-connection test offers a straightforward approach for any microelectrode array subjected to PEDOT deposition or any conductive polymer whose properties change beyond a certain potential threshold. Moreover, the potential step experiment has, for the first time, provided direct insights into the impact of the choice of polymer dopant on recording signals, and the pilot in vivo experiment also indicates the biocompatibility of both PEDOT coatings. This paper lays a foundational framework and introduces robust, broadly applicable techniques for the development and functionality of future neural interface devices.

## Supporting Information Available

Macroelectrode studies on PEDOT deposition conditions; microwire characterization; tetrode fabrication, coating, and cross-connection check; potential step experiment data smoothing, data fitting details and figures of PEDOT:Cl potential step experiment results; in vivo experiment details (PDF)
