# Supplementary material for: Double Electrode Experiments Reveal the Processes Occurring at PEDOT-Coated Neural Electrode Arrays
Source: ACS Appl Mater Interfaces. 2024 May 22;16(22):29439–52. doi: 10.1021/acsami.4c05204 (PMC11163409; doi:10.1021/acsami.4c05204)
Supplement: Supplementary file 1 — am4c05204_si_001.pdf [file am4c05204_si_001.pdf]

Supporting Information:

Double Electrode Experiments Reveal the Processes Occurring at PEDOT-Coated Neural Electrode Arrays

*Yuanmin Zhang[a,b], Yuqi Chen[b], Sonia Contera[a *], and Richard G. Compton[b *]*

[a] Clarendon Laboratory, Department of Physics, University of Oxford, Parks Road, Oxford, OX1 3PU, Great Britain.

[b] Physical and Theoretical Chemistry Laboratory, Department of Chemistry, University of Oxford, South Parks Road, Oxford, OX1 3QZ, Great Britain.

Corresponding Author

*Email: Richard G. Compton: richard.compton@chem.ox.ac.uk

*Email: Sonia Contera: sonia.antoranzcontera@physics.ox.ac.uk

Section 1: Macroelectrode studies on PEDOT deposition conditions

In this section, the electrodeposition of poly(3,4-ethylenedioxythiophene) chloride (PEDOT:Cl) on a platinum (Pt) macro disc electrode is systematically studied and characterized using cyclic voltammetry (CV). This includes studies of the Pt electrode immersed in a monomer solution (EDOT/NaCl) and in solutions without the monomer (NaCl, Na_2SO_4, PBS) to understand the deposition process and the potentials at which identified reactions occur. The electrochemical behavior of the Pt macroelectrode in EDOT/NaPSS and NaPSS has been reported in our previous work [1] and is not repeated here.

1.1 Pt electrode in background electrolyte only (no monomer): NaCl, Na_2SO_4, PBS

Experimental procedure

All electrochemical characterizations were carried out using a three-electrode cell setup, which comprised a Pt macro disk (area = 0.02 cm^2) as the working electrode, a SCE as the reference electrode, and a graphite rod as the counter electrode. Electrodes were first immersed in 0.1 M NaCl, and a wide CV scan was conducted ranging from -0.8 V to E_{max}, where E_{max} varied from an open circuit potential (OCP) to 2.0 V (vs. SCE) at a scan rate of 50 mVs^{-1}. To compare the effect of Pt in solutions with different chloride ion concentrations, the experiment was repeated in 0.01 M PBS and 0.5 M Na_2SO_4. A comparison of their ionic concentrations is presented in Table S1. Note that PBS has a significant chloride concentration.

Results and discussion

It has been reported that Pt itself may undergo oxidation [2, 3], or chloride ions may be oxidized at a Pt electrode [2, 4, 5]. Therefore, before placing Pt in the coating solution (EDOT/NaCl), it is

essential to first determine if any Pt based reactions occur within our potential range of interest and, second, if reactions occur, to identify the products and understand the underlying reactions.

To start with, the Pt macroelectrode was immersed in 0.1 M NaCl. An initial CV scan was made which began at 0 V (vs. OCP), swept anodically to E_{max} of 2.0 V (vs. SCE), then cathodically to -0.8 V (vs. SCE), and finally returned to OCP. Starting the sweep at 0 V (vs. OCP) ensures that no Faradaic reaction occurs at the start of the scan until any higher potential is reached. In Figure S1(a, b), an anodic peak (P1) is observed at approximately 1.35 V, and two cathodic peaks (P1' and H) appear around 0.9 V and 0.1 V, respectively. To further investigate P1, the procedure was repeated with E_{max} values of 0 V (vs. OCP), 1.0 V, and 1.5 V (vs. SCE), as shown in Figure S1(a, b). A comparison of the different E_{max} scans suggests that P1 could correspond to chloride oxidation on the Pt surface. P1' is identified as the reduction peak corresponding to P1 since P1' is absent from the reverse scan when the voltage is lower than the potential of P1 (indicated by the blue and green lines in Figure S1(b)). The peak H was inferred to be due to hydrogen adsorption [3], as it remains even when the CV scan maximum potential is 0 V (vs. OCP) (Figure S1(b), green line).

To further validate the identification of these peaks, the Pt electrode was placed in 0.01 M PBS or 0.5 M Na_2SO_4, respectively, and the above experiments were repeated. The CV scans (PBS in Figure S1(c), Na_2SO_4 in Figure S1(d)) show that all three peaks are observed in PBS when E_{max} is sufficiently high, while only the peak H is present during the reverse scan in Na_2SO_4, regardless of E_{max}. This indicates that the P1 and P1' pair only emerge in the presence of chloride ions and when the potential exceeds 1.35 V. The distinction between the P1 and P1' pair is more apparent when overlaying their CV scans up to 2.0 V (Figure S1(e)), where no significant oxidation is observed in the Na_2SO_4 scan around 1.35 V (Figure S1(d)), and the reduction peak P1' is only seen

in PBS and NaCl. Additionally, overlaying the CV scans up to 1.0 V (Figure S1(f)) reveals a small signal (S1) during the positive scan, which could correspond to Pt oxidation [2, 3].

The reaction of chloride ions at the electrode is described by Equation 1 [2], where E^0 is the standard electrode potential for the reaction. The reaction mechanism for chloride ions on Pt is a two-step process, as suggested by previous studies [2, 4]. Chloride ions are reported to first form a bond with Pt through the removal of one electron (Equation 2), followed by the metal-chloride bond reacting with another chloride ion to regenerate the Pt and form chlorine (Equation 3) [2].

$$2Cl^- \leftrightarrow Cl_2 + 2e^- \qquad (E^0 = 1.359\, V) \tag{1}$$

$$Cl^- + Pt \leftrightarrow PtCl + e^- \tag{2}$$

$$Cl^- + PtCl \leftrightarrow Pt + Cl \tag{3}$$

At potentials lower than 1.0 V, the following reactions happen and lead to the formation of platinum hydroxide and monoxide (Equation 4,5) [2]

$$Pt + H_2O \leftrightarrow Pt - OH + H^+ + e^- \tag{4}$$

$$Pt - OH \leftrightarrow PtO + H^+ + e^- \tag{5}$$

Overall, as supported by the literature [2] and our own experiments, P1 and P1' can be assigned as the peaks for chloride oxidation and its reversal, respectively. The formation of Pt hydroxide and monoxide occurs between 0.5 V and 1.0 V, and this process results in a small shoulder (S1) in Figure S1(f), which is relatively minor compared to the chloride oxidation (P1). Lastly, peak H can be attributed to hydrogen adsorption on Pt, as evidenced by its consistent presence across all CV scans in various solutions (PBS, NaCl, Na_2SO_4).

Table S1: The ionic composition of 0.01 M PBS, 0.1 M NaCl and 0.5 M Na_2SO_4.

Ions	PBS / M	NaCl / M	Na_2SO_4 / M
$Na+$	0.138	0.1	0.5
Cl^-	0.14	0.1	/
K^+	0.0027	/	/
PO_3^{4-}	0.01	/	/
SO_4^{2-}	/	/	0.5

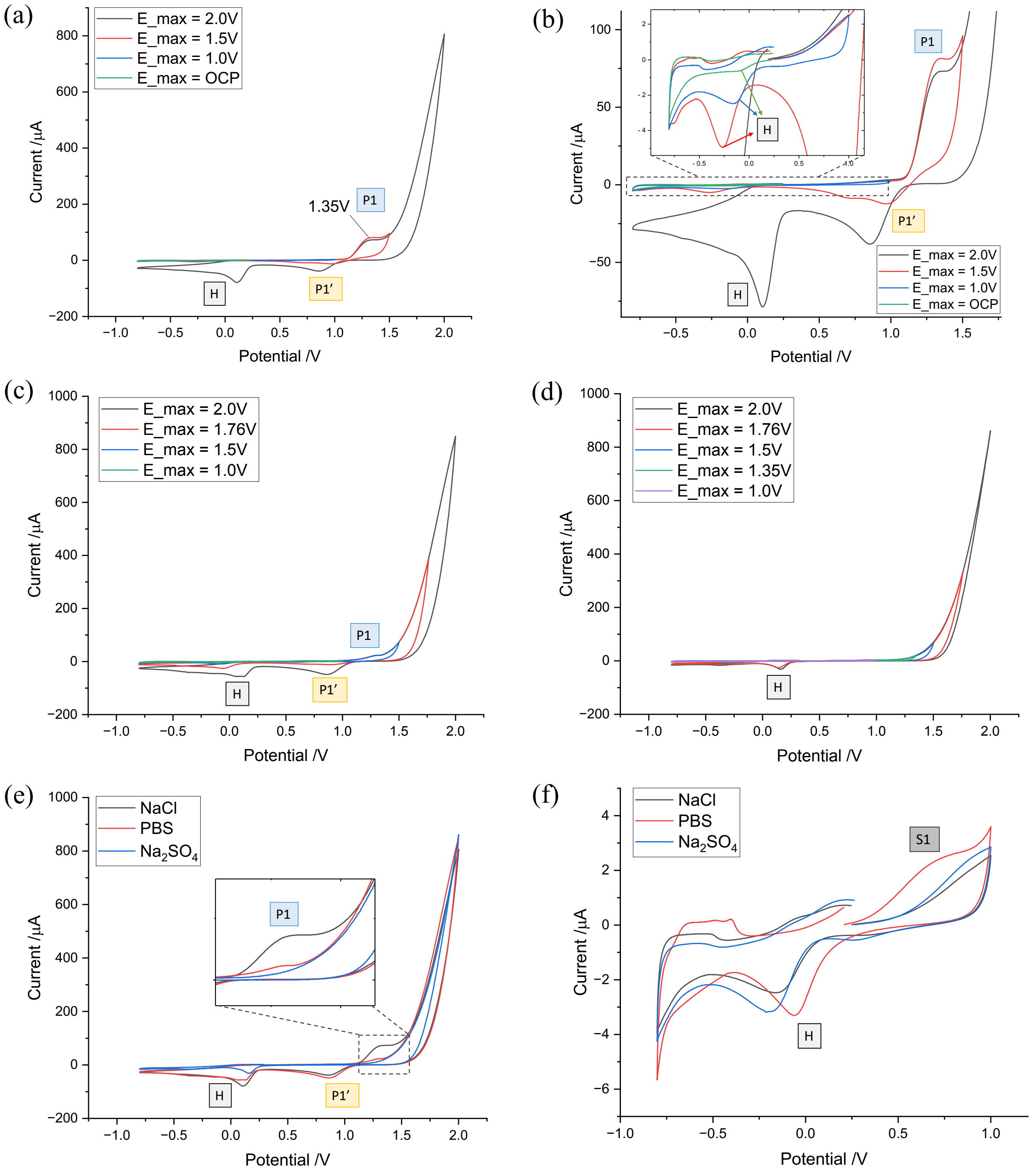

Figure S1: CV scan of bare Pt macroelectrode from the OCP → E_{max} → -0.8V → OCP (scan rate v = 50 mVs^{-1}) **(a,b)** in 0.1M NaCl. (b) is the zoom-in of (a) **(c)** in 0.01 PBS, **(d)** in 0.5M Na_2SO_4. **(e)** Comparison of the CV scan with E_{max} = 2.0V. **(f)** Comparison of the CV scan with E_{max} = 1.0V.

P1, P1': Chloride oxidation and reduction; **H**: Hydrogen adsorption; **S1**: Pt oxidation. OCP is the open circuit potential, which is around 0V vs. SCE. All potentials are reported relative to the SCE.

1.2 Voltammetry of EDOT/NaCl at a Pt Electrode

After identifying the potential where Pt is reactive with chloride in the solution as reported above, CV scans of Pt in EDOT/NaCl were conducted to understand the electrodeposition process in aqueous media.

Experimental procedure

The same electrode cell setup as described in Section 1.1 was used. A bare Pt macroelectrode was immersed in a solution containing 10 mM EDOT and 0.1 M NaCl, and a wide CV scan was performed within the same potential window (-0.8 V to various E_{max}) at a scan rate of 50 mVs^{-1}. Following this PEDOT:Cl was potentiostatically deposited on the Pt electrode at various potentials for a duration of 120 s. The potentials for deposition were chosen based on the prior CV scan results of the bare Pt electrode in the monomer solution. These modified Pt electrodes were transferred to a 0.1 M NaCl solution containing no EDOT, and a CV scan was conducted from -0.8 V to 2.0 V (vs. SCE) at a scan rate of 50 mVs^{-1}. This step was carried out to verify whether the polymer PEDOT:Cl was deposited onto the Pt disc; the presence of a polymer is indicated by a peak attributable to $PEDOT^{+}$ overoxidation, at a potential of ca 1.2 V as demonstrated in our previous work [1]. In the absence of polymer deposition, or if the polymer has already been overoxidized during the deposition process, no corresponding peak is observed during the anodic scan.

Results and discussion

A CV scan was first conducted with a Pt macroelectrode in a solution of 10 mM EDOT and 0.1 M NaCl, starting from OCP scanning up to a maximum potential of 2.0 V (vs. SCE) and then reversing to a potential -0.8 V (vs. SCE). In this way, it was possible to identify potential(s) where electrode reactions happen and hence deduce the potential for electro-polymerization. The CV is shown in Figure S2(a), where two peaks, P1 and P2, can be observed at 1.53 V and 1.21 V, respectively. P1 is assigned to chloride oxidation at Pt with a peak potential of 1.53 V which compares to ca 1.35 V seen in pure 0.1 M NaCl (Figure S1 (a). It is inferred that the presence of the polymer layer slows the reaction. P2 is therefore assigned to a mixture of EDOT oxidation and PEDOT overoxidation of PEDOT. These two reactions are not resolved in EDOT/NaPSS solution [1], but in EDPT/NaCl they merge into a single peak.

To confirm whether P2 is indeed due to mixture of EDOT oxidation and subsequent over-oxidation, the electrode was cleaned, and the same experiment repeated but with various E_{max} values below 1.5 V (that is below P1). The results of these CV scans are overlaid in Figure S2(b). Evidence of over-oxidation at P2 (1.21 V) is indicated by the presence of a P2' reduction peak, observable only when E_{max} exceeds the P2 peak value (Figure S2(b), black line). The E_{max} of 1.35 V was intentionally chosen as it was the previously observed potential for chloride ion oxidation (Figure S1(a)). If chloride oxidation were occurring, a corresponding reduction peak would be expected around 0.9 V during the cathodic sweep. However, no such reduction is observed, further suggesting that the oxidation peak P2 is due to EDOT oxidation and overoxidation rather than a chloride reaction. Note that the peak P2" seen in the range of -0.1 V to -0.2 V is a reduction peak which is present for all E_{max} values equal to or greater than 1.0 V, as shown by the black, green, and red lines in Figure S2(b). Although P2" is too small to be conclusively characterized, it

suggests, even if it is associated with the polymer reduction that the majority of the oxidation process is irreversible and implies that the oxidation of EDOT occurs around 1.0 V to 1.1 V, aligning with our previous experiments [1]. More significantly, this indicates that for PEDOT subjected to scanning at potentials below those needed for over-oxidation (< 1.2 V), there is little, if any, sign of any reduction during the reverse scan. Thus, the oxidation of EDOT in NaCl results in the formation of PEDOT:Cl ($PEDOT^+$ with the dopant Cl^-), which does not undergo reduction on the reverse scan. In essence, no PEDOT is formed with the expulsion of the chloride on the reductive scans.

Next, different coating potentials were selected based on the peak values (P1, P2 = 1.21 V, 1.53 V) observed in Figure S2(a) and previous knowledge of PEDOT oxidation at 1.0V [1]. Potentiostatic deposition was performed, and the results are shown in Figure S2(c). When the modified Pt electrodes were immersed in a monomer-free solution (0.1 M NaCl), only the potentiostatic deposition at 1.0 V displayed an overoxidation peak at approximately 1.25 V (Figure S2(d), red line). It was noticed that for all modified electrodes, no peak was observed around 1.35 V, which corresponds to chloride oxidation on bare Pt. This suggests the presence of a polymer layer, either PEDOT (E = 1.0 V) or over-oxidized PEDOT (E = 1.21 V and 1.53 V), covering the Pt surface and inhibiting the chloride oxidation.

The above experiment indicates that the potential for EDOT/NaCl electro-polymerization occurs around 1.0 V to 1,1V. Beyond 1.2 V (P2), the reaction forms a mix of PEDOT and over-oxidized PEDOT. Chloride oxidation is not a concern at this stage, as it is shifted to a higher potential of around 1.5 V due to the presence of the polymer layer. Once covered by the polymer, chloride ions cannot as easily access the Pt substrate as compared to a bare electrode, and

consequently, no chloride oxidation peak is observed, which could otherwise be confused with the over-oxidation peak of PEDOT.

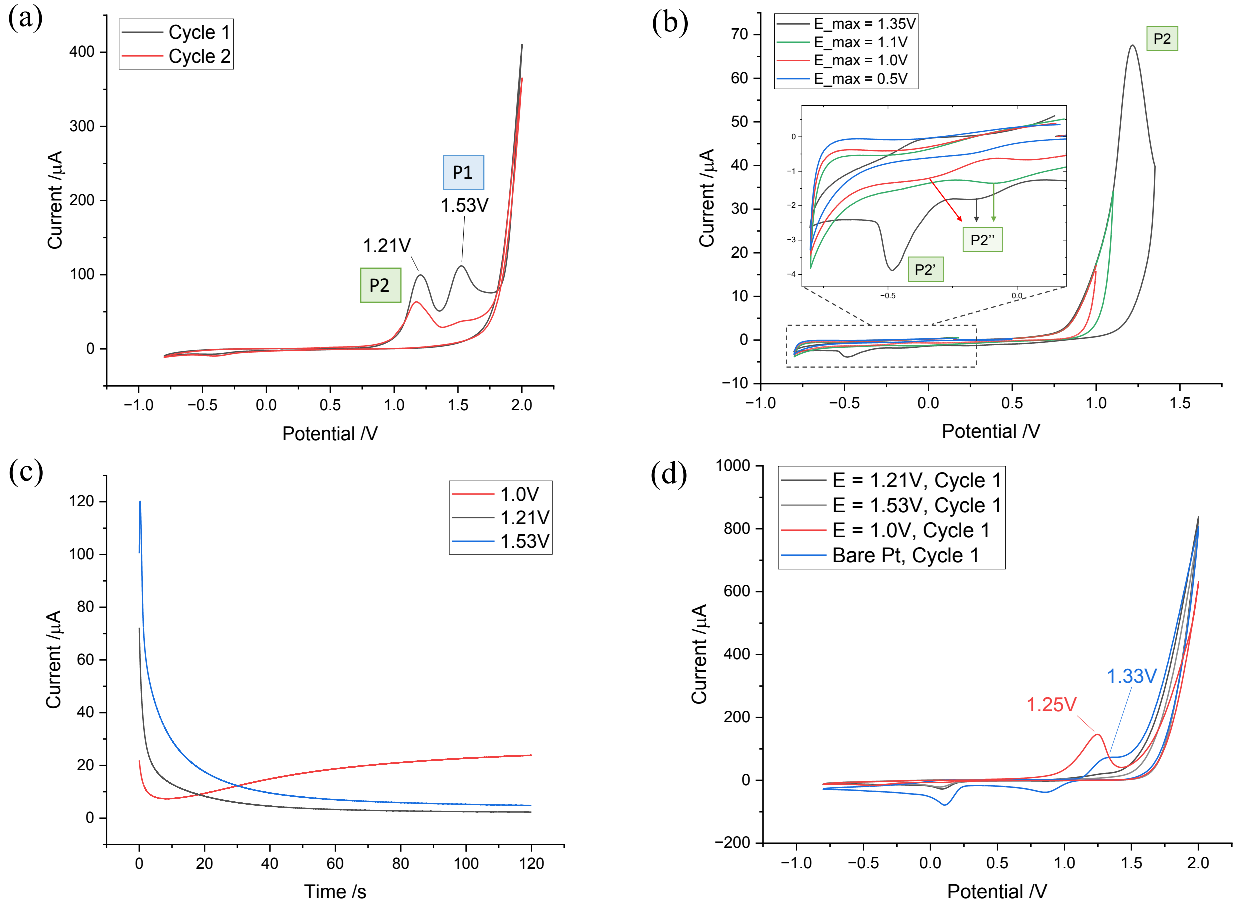

Figure S2 (a, b) CV scan of a bare Pt macroelectrode from OCP → E_{max} → -0.8V → OCP in 10 mM EDOT and 0.1 M NaCl (scan rate v = 50 mVs^{-1}). E_{max} = 2.0 V for (a) and 0.5 V ~ 1.35 V for (b). **(c)** Chronoamperograms using the same electrode and solution were recorded at potentials of E = 1.0 V, 1.21 V, and 1.53 V for 120 s. **(d)** CV scan of modified and bare Pt electrodes in 0.1 M NaCl, scanning from OCP → 2.0 V (vs. SCE) → -0.8 V (vs. SCE) → OCP (scan rate v = 50 mVs^{-1}). All potentials are reported relative to the SCE.

Section 2: Microwire characterization

In this section, the microelectrodes used are systematically studied and characterized using cyclic voltammetry (CV). Individual tungsten (W) and platinum (Pt) microwires are characterized using CV in solutions with and without the monomer EDOT present to understand their electrochemical processes. Subsequently, electropolymerization is carried out on Pt microwires, with the resulting coatings characterized and compared with previous macroelectrode findings. More importantly, the electropolymerization conditions established were subsequently applied to the coating of tetrodes. Additionally, a new and straightforward testing method for polymer overgrowth and checking for any cross-connections between electrodes within a tetrode will be introduced.

2.1 W microwire CV scan in solution with and without monomer

Tungsten (W) has frequently been used for implanted electrodes [6, 7]. However, it has been reported that tungsten oxidation and resulting dissolution products are toxic and compromise the long-term viability of the implants [8]. Therefore, it is useful to see if tungsten can be coated with PEDOT without triggering the formation of any oxides and subsequent corrosion, thereby avoiding the production of toxic products and enhancing the device longevity in implant applications.

Experimental procedure

All electrochemical characterizations were conducted using a three-electrode setup, as in section 1, except that the working electrode was a tungsten (W) microwire (d = 12.7 μm) coated with an insulation layer of Heavy Formvar (HFV) around its body. To connect to the Autolab, the insulation on one end of the microwire was gently removed by using a new, sharp scalpel. After the insulation layer was peeled off, the shiny metal underneath was directly observed with the naked eye. The exposure of the metal was confirmed by microscope. The exposed end of the metal

wire was then connected to a thicker and longer stainless-steel wire using silver epoxy (RS Components Ltd, UK), as depicted in Figure S3 B and C. The stainless-steel wire was inserted through a pipette head first, where the latter was used to stabilize the wire and protect the wire body from bending and scratching when the experiments were carried out. The end of the stainless-steel wire (Figure S3 C) could then be easily connected to the electrochemical apparatus. The end of the W microwire (Figure S3 A) was cut with fine scissors (14568-12, German Stainless) before each experiment. Before starting experiments, a CV scan from 0.5 V to 1.0 V in a solution, such as 0.01 M PBS, was performed to test the wire connection.

Electrodes were immersed in solutions containing monomer (10 mM EDOT and 0.1 mM NaPSS) and without monomer (0.1 mM NaPSS), respectively. A CV scan was carried out, starting from OCP to 1.7 V (vs. SCE) and -0.8V (vs. SCE) at a scan rate of 50 mVs^{-1} and continuing for 5 successive cycles.

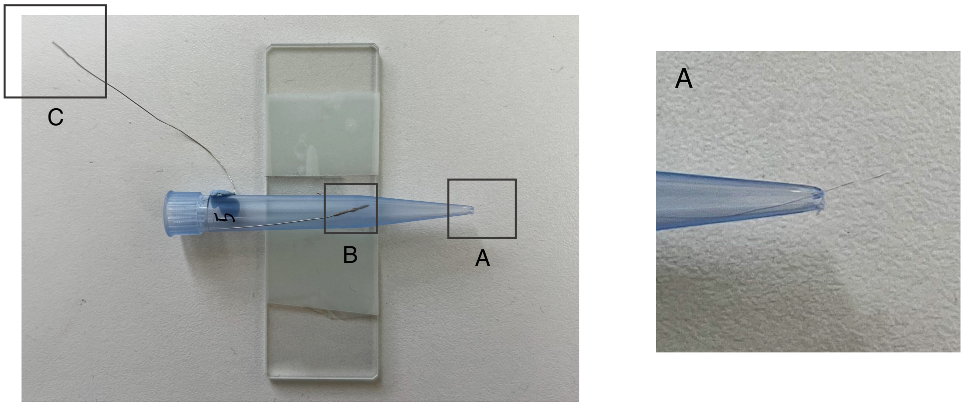

Figure S3: Setup for a microwire connection. **Box A:** Microwire end to be immersed into the solution (Zoom in is shown on the right). **Box B:** The connection between the stainless-steel wire and the microwire using silver epoxy. **Box C:** Stainless steel wire that can be clamped onto with a crocodile clip and connected to the electrochemical apparatus.

Results and discussion

A CV scan from 0.5 V to 1.0 V in 0.01 M PBS was carried out to test the wire connection. A bad connection will result in a purely noisy current fluctuating around 0 A (Figure S4(a); a good connection, however, will exhibit a much higher and stable current (Figure S4(b)).

To characterize the W microwires, a CV scan was recorded starting from OCP to 1.7 V (vs. SCE), then reversed to -0.8 V (vs. SCE), and finally returned to the initial OCP value in a solution containing both 10 mM EDOT and 0.1 mM NaPSS, as well as in a solution with only the background electrolyte 0.1 mM NaPSS. From Figure S4(c, d), both scans exhibit a peak around 0.5 V (P_w1) during the initial cycle, which then shifts to approximately 0.9 V (P_w1') in subsequent cycles. P_w1 and Pw1' are assigned to tungsten oxidation. These two peaks appear at similar potentials and peak heights in both scans, regardless of the presence of the monomer EDOT. In contrast, P_w2 (ca 1.5 V) is only present in the scan in EDOT/NaPSS (Figure S4(c)), indicating it is a peak resulting from a combination of EDOT oxidation and PEDOT overoxidation, as discussed below. The merging of EDOT and PEDOT oxidation reactions is clearer when comparing the CV scans in EDOT/NaPSS using Pt microwires of different sizes in section 2.2.

Therefore, unlike the previous results with Pt in EDOT/NaCl (Section 1.2), where chloride oxidation on Pt only occurs at potentials beyond polymer formation, W oxidation appears to occur prior to polymer deposition and continues post-polymer formation. The current generated by such oxidation (P_w1) is not negligible compared to the polymer oxidation peak (P_w2), suggesting that the product might be a mixture of W oxides and PEDOT. Previous studies have suggested several possible reactions at the W surface (Equation 6, 7) [8-10], but the precise electrochemical reactions remain unclear [8].

$$W + 4H_2O \rightarrow WO_4^{2-} + 8H^+ + 6e^- \quad (6)$$

$$WO_3 + H_2O \rightarrow WO_4^{2-} + 2H^+ \quad (7)$$

Moreover, the challenge of preventing oxidation cannot mitigated by PEDOT deposition. Consequently, subsequent sections will focus on Pt, a better candidate for implant microelectrode [8] and for surface modification, where no oxidation except for polymer formation is expected within the potential range of interest.

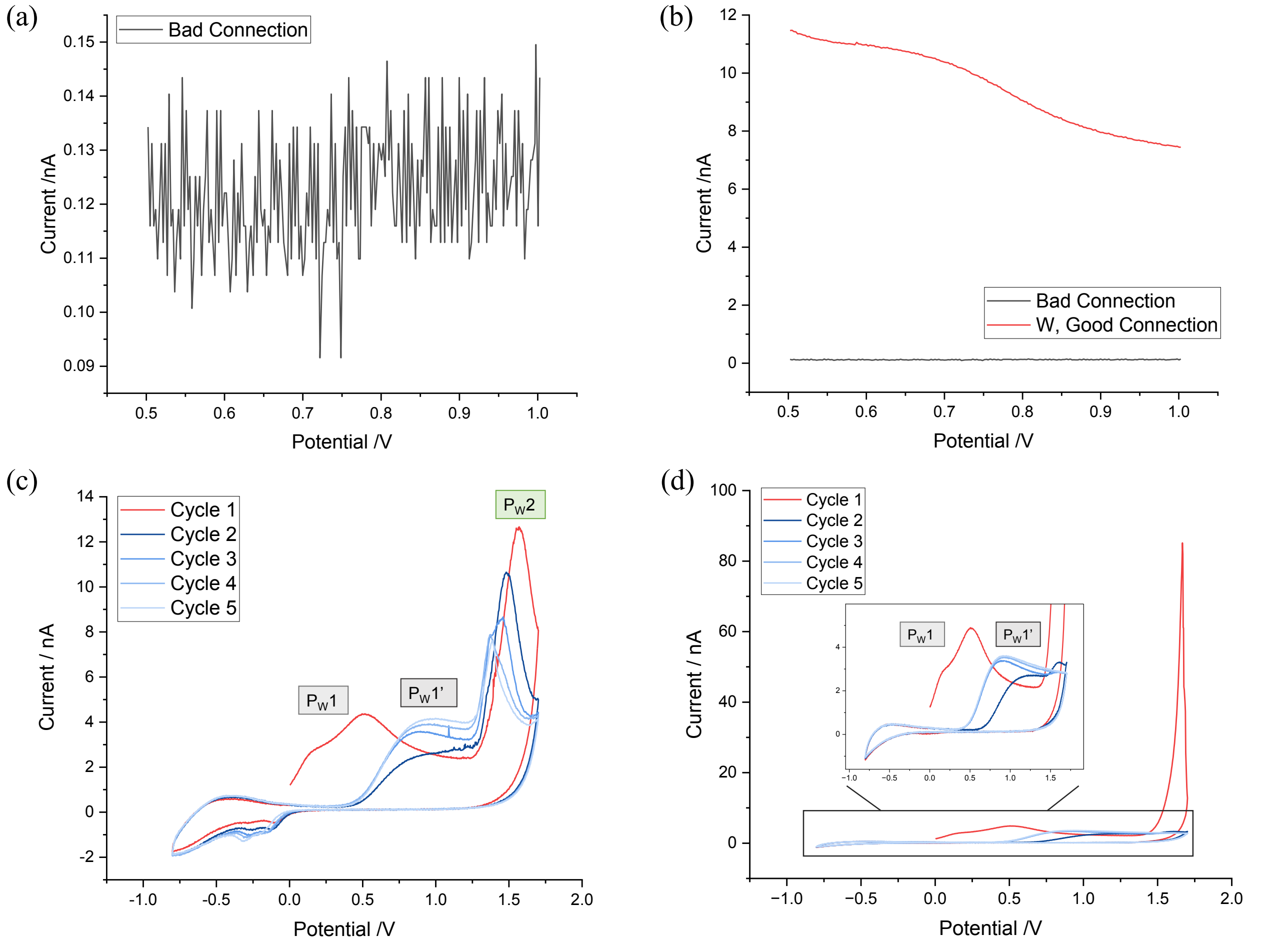

Figure S4: (a) Example CV for a "bad" W wire connection. **(b)** Contrast between "good" and "bad" connections to W wires. **(c~d)** CV scan of bare W microwire (d = 12.7 μm) from the OCP → 1.7 V → -0.8V → OCP **(c)** in 10 mM EDOT and 0.1 mM NaPSS. **(d)** in 0.1 mM NaPSS. **P_w1,**

P_W1': W oxidation. **P_W2:** Mix of EDOT oxidation and PEDOT overoxidation. Scan rate v = 50 mVs^{-1} for all the above scans. All potentials are reported relative to the SCE.

2.2 EDOT voltammetry at Pt microwire electrodes

In this section, we first explore via CV the effect of different Pt electrode sizes on the electro-oxidation of EDOT in NaPSS, as detailed in section 2.2.1. Building on previous experiments[1] conducted with a Pt macroelectrode, we study Pt microelectrodes of two different diameters and identify the electrochemical reactions occurring so as to deduce better coating conditions. Subsequently, a galvanostatic deposition is performed on Pt microelectrodes. The coatings are characterized in the background electrolyte containing only NaPSS, and the results compared with those from the macroelectrode, particularly focusing on the charge associated with polymer overoxidation.

After testing with EDOT/NaPSS, the Pt microelectrode is used to explore the EDOT/NaCl in section 2.2.2. Galvanostatic deposition conditions for EDOT/NaCl are also examined. Finally, the results of different polymer coatings (PEDOT:PSS and PEDOT:Cl) are compared.

2.2.1 Pt microwire in EDOT/NaPSS

Experimental Procedures

The setup as described in section 2.1 was used, except for substituting the W microwire with Pt microwire electrodes. Two different diameters of Pt microelectrodes were employed with diameters (d) 15 and 25 μm. Both wires were coated with polyimide insulation, which was removed on one end using a sharp scalpel, following the procedures outlined in section 2.1. The

final microwire connection was made exactly as depicted in Figure S3, and the wire connection was verified with a CV scan as above. It should be noted that the 15 μm microwire was only used to compare the effect of wire size on the CV scan in EDOT/NaPSS, as it has a radius comparable to the previously used W microwire (d = 12.7 μm), making it useful for comparing and understanding the electrochemical processes at this size scale. However, since Pt is softer compared to W and such thin and soft wires are known to be easily bent during implantation surgery causing unwanted challenges, it was not utilized for further tests on galvanostatic deposition or implantation. Thus, most comparisons between macro and microwires were made using the 25 μm diameter wires.

A bare Pt microelectrode was immersed in a solution of 10 mM EDOT and 0.1 mM NaPSS, and a CV scan was conducted from OCP to 2.0 V (vs. SCE), and then to -0.8 V (vs. SCE). This procedure was repeated for both sizes of Pt microwires. Subsequently, the 25 μm diameter microwire was cut to expose fresh Pt, and placed into a solution containing only the background electrolyte of 0.1 mM NaPSS. The outcomes of these scans were compared with those obtained using a macroelectrode to, first, understand the electrochemical processes occurring and, second, deduce the optimal coating conditions for the microelectrode. In addition, it needs to be noted that the dopant ratio is not affected by the ratio of materials in the polymerization solution. Previous studies suggest that EDOT oxidizes more readily in solutions with higher concentrations of NaPSS [11]. For instance, the oxidation potential is 1.05 V for 0.001 mM NaPSS but decreases to 0.95 V and 0.90 V for 0.01 mM and 0.1 mM NaPSS, respectively. This is because the positively charged $PEDOT^+$ chains require negative counterions to balance the charge.

A galvanostatic deposition was carried out using a current of 20 nA for 13 s on the 25 μm Pt microelectrode. The conditions were chosen to maintain the deposition charge density at

approximately 50mCcm^{-2}, consistent with findings from previous macroelectrode studies[1]. Following this, the modified Pt microwire was immersed in a 10 mM NaPSS solution to conduct a CV scan ranging from OCP to 1.35 V (vs. SCE) and then to 0 V (vs. SCE).

Results and discussion

The voltammograms recorded at both Pt microwires in the monomer solution from OCP to 1.7 V and back to -0.8 V. These results are presented together with previously reported macroelectrode data[1] in Figure S5(a). The major oxidation peak (P) shifts more negative potentials as the dimensions of the wire get smaller. This might be caused by the different diffusion to and from micro- and macro-disk electrodes with the greater current density seen at microelectrodes leading to the more rapid completion of the process. A shoulder (S) was be observed beside the major peak for Pt (d = 25 μm) (Figure S5(a)), similar to what is seen for the macroelectrode[1], where the shoulder (S) represents EDOT polymerization, and the major peak (P) is the PEDOT overoxidation[1]. The shoulder is not visible for the smaller Pt wire (d=15 μm) due to the shift of P with potential. The observations help explain why in the case of W microwire (d = 12.7 μm), the P_w2 in Figure S4(c) is concluded to be a combination of EDOT polymerization and PEDOT overoxidation.

Next CV scans of bare 25 μm Pt micro and macro electrodes in 0.1 mM NaPSS were conducted and are displayed in Figure S5(b, c). The current density in the microelectrode scan (Figure S5(b)) is significantly higher than that in the macroelectrode (Figure S5(c)), as anticipated. However, the major electrochemical reactions remain consistent; both electrode types exhibit Pt oxide formation within the potential range of 0.5 V to 1.0 V vs. SCE and reduction around 0.0 V vs. SCE. Based on the CV results, we transitioned to using the galvanostatic deposition method instead of

potentiostatic deposition to prevent PEDOT overoxidation while ensuring that the charge deposition per unit area remains constant.

The choice of galvanostatic deposition maintained the same current density as that used in the macroelectrode deposition ($\sigma_{ave} = 50\ mCcm^{-2}$). The monomer oxidation on the macroelectrode was observed at a potential of ca 1.0V with a current of ca. 80 μA[1]. Therefore the current density during oxidation (J_{ox}) can be calculated as in the following (Equation 8) which can then be employed to determine the current (I_{dep}) and duration (t_{dep}) for deposition on the 25 μm microelectrode (Equation 9, 10):

$$J_{ox} = \frac{I_{ox}}{A_{macro}} = \frac{80\mu A}{0.02cm^2} = 4mAcm^{-2} \tag{8}$$

$$I_{dep} = J_{ox} \times A_{micro} = 4mAcm^{-2} \times \left(\frac{25\times10^{-4}cm^{-2}}{2}\right)^2 \times \pi \approx 20\text{nA} \tag{9}$$

$$t_{dep} = \frac{\sigma_{ave}}{J_{ox}} = \frac{50mCcm^{-2}}{4mAcm^{-2}} \approx 13s \tag{10}$$

The galvanostatic deposition was applied to a Pt microwire (d = 25 μm) with a current of 20 nA for 13 s, as shown in Figure S6(a), resulting in a final charge density of around 50 $mCcm^{-2}$. The deposition was repeated for three independent trials, and the resulting deposition curves demonstrated a high level of consistency.

The coated Pt microwire was then placed in 0.1 mM NaPSS for a CV scan starting from OCP and up to 1.35 V (vs. SCE) and reversed back to -0.8 V (vs. SCE) for successive 3 cycles. The results are overlaid with the macroelectrode scans, ranging from OCP to 1.7 V (vs. SCE), as shown in Figure S6(b). These potential ranges were chosen to capture the entire overoxidation peak without reaching a potential that is high enough to cause solvent breakdown.

As illustrated in Figure S6(b), both the micro and macro electrodes exhibit an overoxidation peak in the first cycle, with no peak observed in subsequent scans due to the destruction of PEDOT electrical properties from overoxidation[1, 12, 13], preventing further electrochemical reactions. Notably, a shift in the overoxidation peak to less positive potentials occurs, analogous to the shift of the major oxidation peak observed in Figure S5(a) as the electrode size decreases. Further, the first cycle scans of the micro and macro electrodes were extracted and are presented in Figure S6(c) and Figure S6(d), respectively with the aim of comparing the charge transferred during the overoxidation process to approximately estimate the amount of polymer deposited. After baseline subtraction (indicated by the red lines), the areas under the curves were calculated. The outcomes reveal that the areas of the overoxidation peaks for the micro (Area ≈ 3.2 $mQcm^{-2}$) and macro (Area ≈ 2.8 $mQcm^{-2}$) electrodes are comparable, suggesting that the degree of overoxidation is similar and that the intended charge density deposition was successfully achieved.

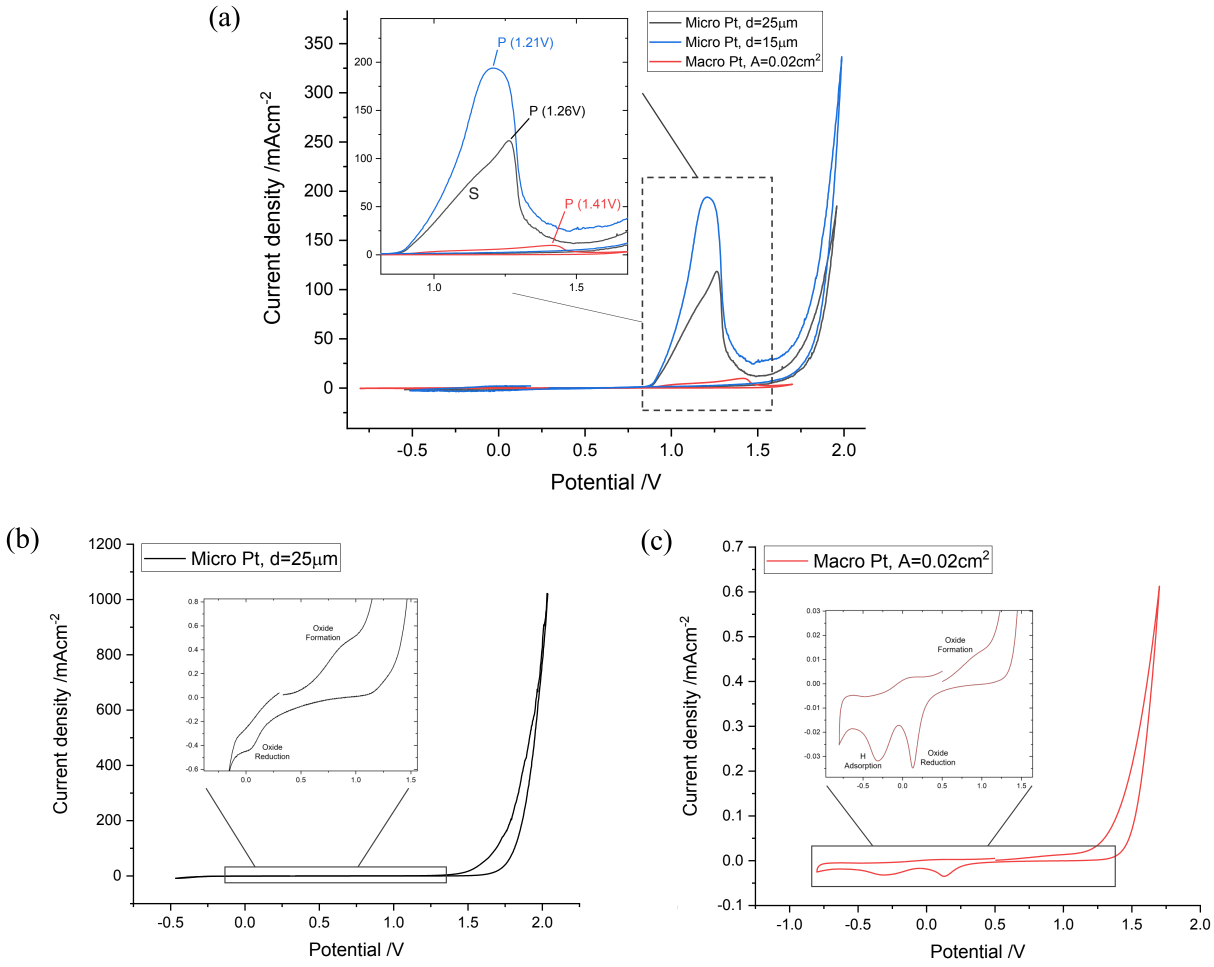

Figure S5: (a) CV scan from OCP→ 2.0V → -0.5V → OCP was conducted for Pt microwires of d = 25 μm and 15 μm in solution containing 10 mM EDOT and 0.1 mM NaPSS. The results are overlayed with the previous macroelectrode CV scan, which ranged from -0.7 V to 1.7 V. **(b)** CV scan from OCP → 2.0V → -0.5V → OCP using Pt microwire with d = 25 μm, **(c)** CV scan from OCP → 2.0V → -0.8V → OCP using Pt macroelectrode with area A = 0.02 cm^2. Scan rate v = 50 mVs^{-1} for all the above scans. All potentials are reported relative to the SCE.

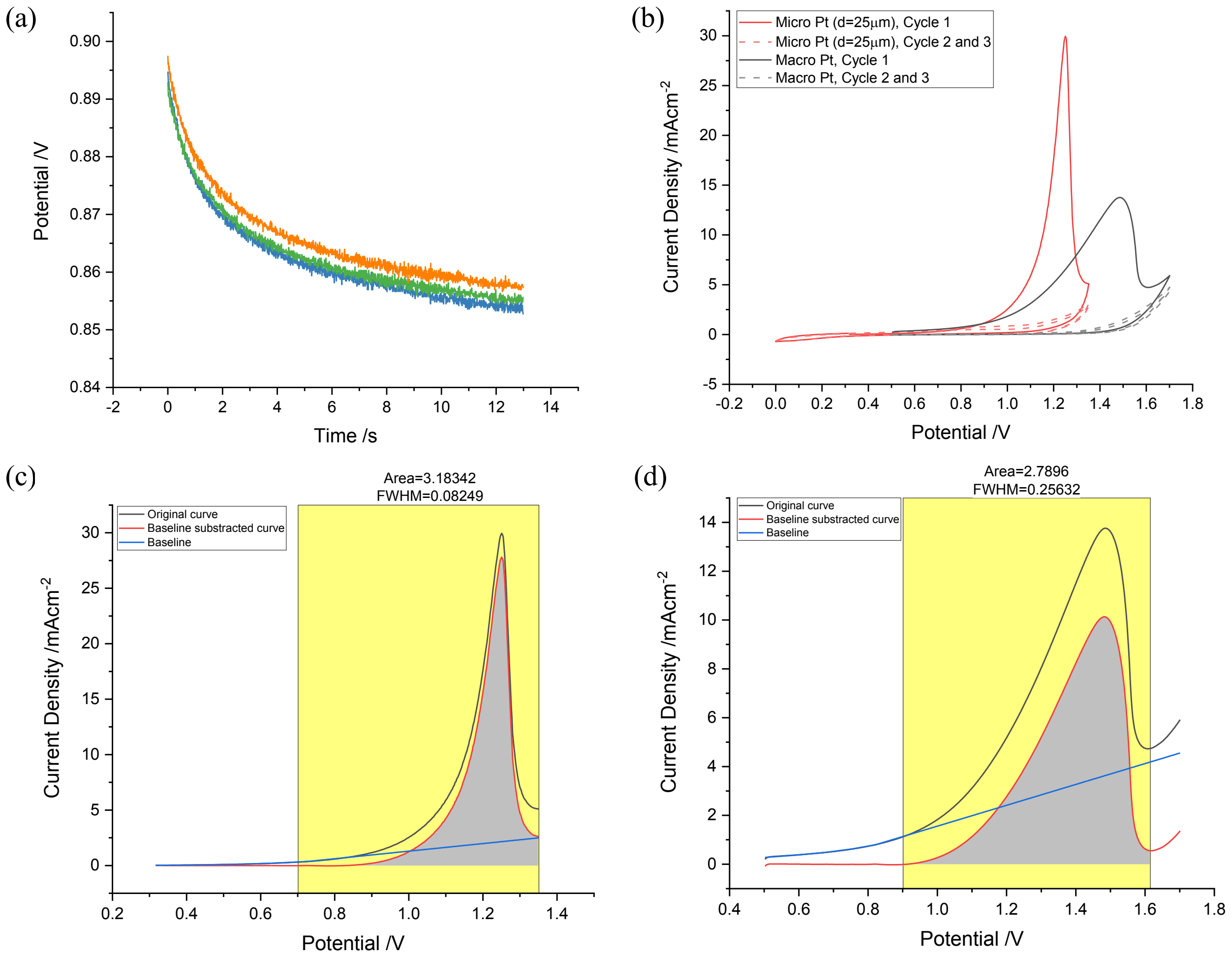

Figure S6: (a) Galvanostatic deposition of three independent trials. **(b)** On microelectrode, CV scans from OCP → 1.35V → 0V → OCP. On macroelectrode, CV scans from OCP →1.7V →OCP. **(c)** The area under the microelectrode overoxidation peak (Area ≈ 3.2 mQcm^{-2}), **(d)** The area under the macroelectrode overoxidation peak (Area ≈ 2.8 mQcm^{-2}). Scan rate v = 50 mVs^{-1} for all the above CV scans. All potentials are reported relative to the SCE.

2.2.2 Voltammetry of EDOT/NaCl at a Pt electrode

Experimental Procedures

The same setup as described in 2.2.1 was employed using 25 μm diameter Pt electrodes.

A bare Pt microelectrode was first immersed in a solution of 0.1 M NaCl, where a CV scan was conducted from OCP to 1.7 V (vs. SCE), and then to -0.8 V (vs. SCE). This procedure was repeated for solutions of 0.01 M PBS, 0.5 M Na_2SO_4, and 10 mM EDOT with 0.1 M NaCl, respectively, to evaluate the effects of different chloride concentrations and the presence of EDOT monomer on the Pt microelectrode.

Subsequently, to optimize the deposition conditions, a range of fixed currents ranging between 15 nA and 28 nA was chosen for the galvanostatic coating in 10 mM EDOT and 0.1 M NaCl. The experiment was repeated in a solution containing only 0.1 M NaCl to assess whether the change of potential was due to chloride oxidation or EDOT oxidation.

Following the optimization steps, a coating condition of 20 nA for 13 s was selected to be consistent with the previous method on PEDOT:PSS. The PEDOT:Cl-coated Pt microwire was then immersed in 0.01 M PBS to test the coating by searching for any PEDOT overoxidation peak with a CV scan spanning from 0 V to 1.5 V (vs. SCE) over 3 successive cycles. For comparison, the Pt microwire was then cut and coated with PEDOT:PSS using the same conditions as before (20 nA, 13 s) and this modified microwire underwent the same CV scan in 0.01 M PBS for 3 cycles. For comparison, the Pt microwire was subsequently cut and coated with PEDOT:PSS using the same conditions (20 nA, 13 s), and this modified microwire was subjected to the same CV scan in 0.01 M PBS for 3 cycles

Results

The CV scans of the bare Pt microwire in 0.1 M NaCl, 0.01 M PBS, 0.5 M Na_2SO_4, and 10 mM EDOT with 0.1 M NaCl are summarized in Figure S7(a). As seen in Figure S7(a), peak P2 (ca 1.21 V) represents PEDOT overoxidation, while P1 (ca 1.35 V) corresponds to chloride oxidation, similar to the findings discussed with the macroelectrode in Section 1.1. The difference in the scale of P1 in non-EDOT solutions compared to Figures S1(e) and S2(a) is attributed to the higher current densities on the microelectrode compared to the macro disk, which leads to a shorter timescale for completing the process. When comparing scans between the micro and macro electrodes in EDOT/NaCl, the deposition of the polymer quickly covered the small cross-section of the microwire, thereby inhibiting the chloride reaction and resulting in a much smaller P1 in Figure S7(a) compared to Figure S2(a).

Next, a freshly cut Pt microwire was placed in a solution of 10 mM EDOT and 0.1 M NaCl to test the galvanostatic deposition conditions with currents ranging from 15 nA to 28 nA for 20 s. As illustrated in Figure S7(b), similar galvanostatic curves were observed for currents within the 15 nA to 22 nA range. For currents higher or equal to 23nA, the reaction potential increased. In particular, the potential shifted to above 1.3 V, which is near the chloride oxidation potential at the Pt surface. To ascertain whether this increase was related to chloride oxidation, the galvanostatic experiments were repeated in 0.1 M NaCl. The results, illustrated in Figure S7(c), show a consistent increasing trend in potential, implying that this phenomenon could be due to chloride oxidation on the Pt. Thus, a current between 15 nA and 22 nA is more suitable for electropolymerization, especially since the potential in this current range is between 1.0 V and 1.1 V, which corresponds to the range for monomer oxidation alone, as mentioned in section 1.2.

Given the negligible differences when selecting a current between 15 nA and 22 nA, and for consistency with the deposition conditions of PEDOT:PSS (Section 2.2.1), a deposition current of 20 nA for 13 s was chosen for subsequent experiments. The PEDOT:Cl-coated Pt microwire was then immersed in 0.01 M PBS, and the CV was compared with that for PEDOT:PSS-coated Pt microwire. Their galvanostatic curves are shown in Figure S7(d), and their CV scans in 0.01 M PBS are presented in Figure S7(e). From Figure S7(e), overoxidation peaks are observable for both types of polymer-coated electrodes, with their peak positions aligning with each other very well at around 1.2 V, indicating that EDOT was successfully oxidized in both case to form either PEDOT:Cl or PEDOT:PSS on the Pt surface.

Comparison of the micro and macro electrode CV scans in solutions containing with EDOT monomer, reveals much higher current densities at the microelectrode disc. This leads to more rapid completion of electrochemical processes, where the effect is more obvious in smaller diameter electrodes (12.7 μm W and 15 μm Pt) resulting in the merging of oxidation and overoxidation peaks. Consequently, the galvanostatic deposition method was chosen to prevent PEDOT overoxidation, with a current of 20 nA for 13 s being selected to ensure that the charge deposition per unit area achieves an average density of $\sigma_{ave} = 50\, mCcm^{-2}$. Electropolymerization using this method to deposit PEDOT:PSS and PEDOT:Cl on Pt was tested in PBS. The presence of overoxidation peaks in both cases was successfully used to fingerprint successful coating. Thus, in the subsequent section, this galvanostatic deposition condition is maintained and applied to a Pt tetrode.

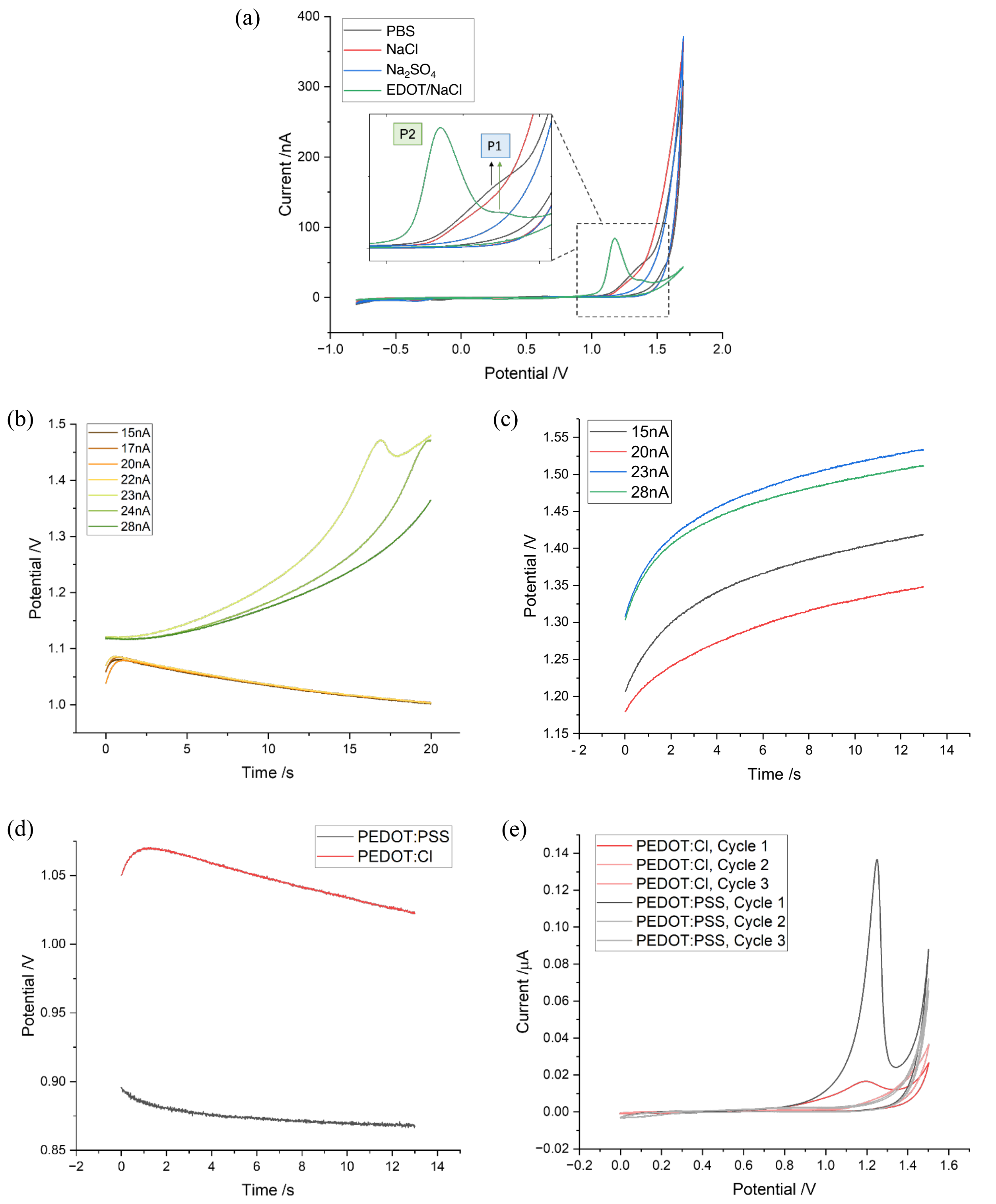

Figure S7: (a) Comparison of the CV scans from OCP → 1.7V →-0.8V → OCP using Pt microelectrode (d = 25 µm) in 0.01 M PBS, 0.1 M NaCl, 0.5 M Na_2SO_4, and 10 mM EDOT with 0.1 M NaCl, respectively. **(b)** Galvanostatic deposition of Pt immersed in 10 mM EDOT and 0.1

M NaCl, holding current at different values for 20 s. **(c)** Galvanostatic deposition of Pt immersed in 0.1 M NaCl, holding current at different values for 13 s. **(d)** Galvanostatic deposition of Pt immersed in 10 mM EDOT and 0.1 M NaCl or 10 mM EDOT and 0.1 mM NaPSS. Coating conditions were both 20 nA for 13 s. **(e)** Three successive CV scans of PEDOT:Cl and PEDOT:PSS coated Pt immersed in 0.01 M PBS for a CV scan OCP →1.5 V → 0 V → OCP. Scan rate $v = 50\ \mathrm{mVs^{-1}}$ for all the above CV scans. All potentials are reported relative to the SCE.

2.3 Pt tetrode fabrication, coating and electrode isolation checking

In Section 2.3.1, we first outline the method for fabricating a tetrode and its characterization using scanning electron microscopy (SEM). In section 2.3.2, we describe the coating of the Pt electrodes within the tetrode with PEDOT:PSS, and the electrical isolation of the electrodes from one another. The coated and uncoated Pt surfaces were further analyzed and compared using SEM, energy-dispersive X-ray spectroscopy (EDX), and optical microscopy.

2.3.1 Tetrode fabrication

Experimental procedures

Tetrode Fabrication: The process is depicted in Figure S8. A microwire of 20 to 30 cm in length was cut based on its intended use and the apparatus to which it was to be connected. Tape was used to fix both ends of the microwire together (Figure S8(a)), with one end (Figure S8(a), S) affixed to a rod attached to a clamp stand. The opposite free end (Figure S8(a), F) was positioned as shown in Figure S8(b). A small magnetic weight, equipped with a hook at the top, was hung at the bottom of the wire in order to stretch four wire segments straight (Figure S8(b)). A magnetic

stirrer plate was positioned beneath the weight to rotate the magnetic weight and twist the wires together at a consistent rate (Figure S8(c)). Before turning on the stirrer, it was ensured that the weight was centered on the stirrer plate to avoid wobbling and possible wire breakage. The twisting was stopped once enough height was achieved, leaving about 2 cm of untwisted wire near the rod. The stirrer plate was then carefully removed, allowing the wire to unwind slightly to alleviate stress, and the stirrer plate was replaced to stabilize the wire and the weight. Without this step, the wire might recoil when the weight is removed, leading to an undesired tetrode shape. A heat gun set to the appropriate temperature was used to melt the wire outer insulation slightly, causing the strands to fuse. For a W microwire insulated with FHV (glass transition temperature at ca. 105˚C [14]), the heat gun was set to 200˚C, while for a Pt microwire insulated with polyimide, which has a higher glass transition temperature (ca. 220˚C [14]), the heat gun was at a temperature of ca. 300˚C. Note that the rod material should be heat-resistant, for example wood or glass, to prevent insulation material from melting onto the rod during the heat-curing process. After cooling the tetrode for a few minutes, sharp scissors were used to cut the end attached to the weight, followed by the top two ends.

To connect to the electrochemical apparatus, the insulation on the four tetrode free ends was removed using a scalpel. Similar to single microwire attachment as described in section 2.1, all four wires of the tetrode were attached to four stainless steel wires (Figure S8(d) C) respectively using silver epoxy. Pipette heads, as shown in Figure S3, were used to hold the stainless-steel wires, protect the connection, and stabilize the microwires. However, before making the connections, four pipette heads were first fixed together by placing a piece of Blu Tack (a moldable, clay-like, and pressure-sensitive adhesive produced by Bostik) to hold them together (Figure S8(d) B), after which stainless steel wires were inserted and connections made with the free ends of a tetrode.

This arrangement of securing the four pipette heads prevents them from moving against each other during operation, which could potentially stretch the microwires and damage the tetrode. The final assembly is shown in Figure S8(d), and the tetrode head detail is illustrated in box A. The setup was allowed to dry overnight before connection testing or use.

Tetrode Inspection: Before initiating the coating process, a small segment from the head of the tetrode (Figure S8(d) A) was carefully cut and affixed to an SEM specimen stub using an adhesive carbon tab. This wire segment was positioned at the edge of the specimen stub to facilitate observation. SEM images were captured for W tetrodes after the heat-curing process, as commonly used before [15]. Images of Pt tetrodes, both with and without heat curing, were similarly recorded.

Results:

The SEM images of the of bare tetrode body and cross-sections are shown in Figure S9. W tetrodes have insulation made of Heavy Formvar (HFV), which can be melted and fused together using a heat gun at 200˚C (Figure S9(a)). In contrast, a Pt tetrode, as shown in Figure S9(c), shows no melting of the polyimide insulation at this temperature. However, after heat curing at approximately 300˚C, the four Pt wires are tightly packed together, as illustrated in Figures S9(d, e). Moreover, the tetrode body does not exhibit significant alterations after heating, as evidenced by comparing Figure S9(e, f) with the non-heated example in Figure S9(b).

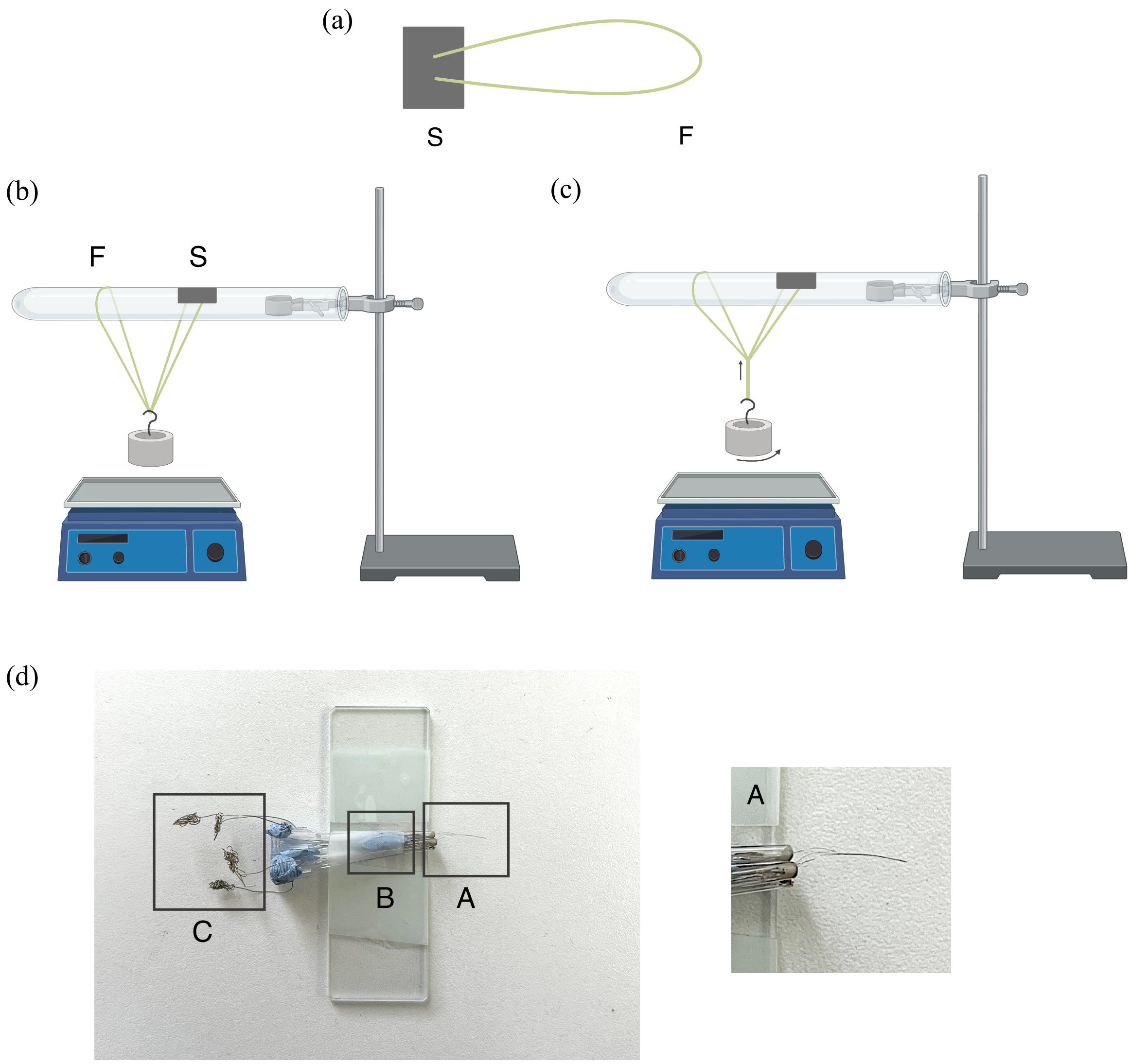

Figure S8 (a) A microwire fixed by tape. S represents the fixed end, and F represents the free end. **(b)** Tetrode fabrication setup. A glass tube is secured to a clamp stand to support the microwire. A magnetic weight, hooked at the bottom, ensures the wire segments are stretched, with a magnetic stirrer plate positioned underneath. **(c)** Turning on the stirrer plate causes the wires to twist into a single bundle. The twisting was stopped once the tetrode reached the desired length. **(d)** Tetrode connection setup. **Box A:** Tetrode end that will be immersed into the solution (Zoom in is shown on the right). **Box B:** Blu Tack (a moldable adhesive, Bostik) is placed in the middle to hold the

pipette heads in place so that they will not slide over each other and break the tetrode. **Box C:** Stainless steel wires that connected via a crocodile clip to the electrochemical apparatus. (a~c) are created using BioRender.com.

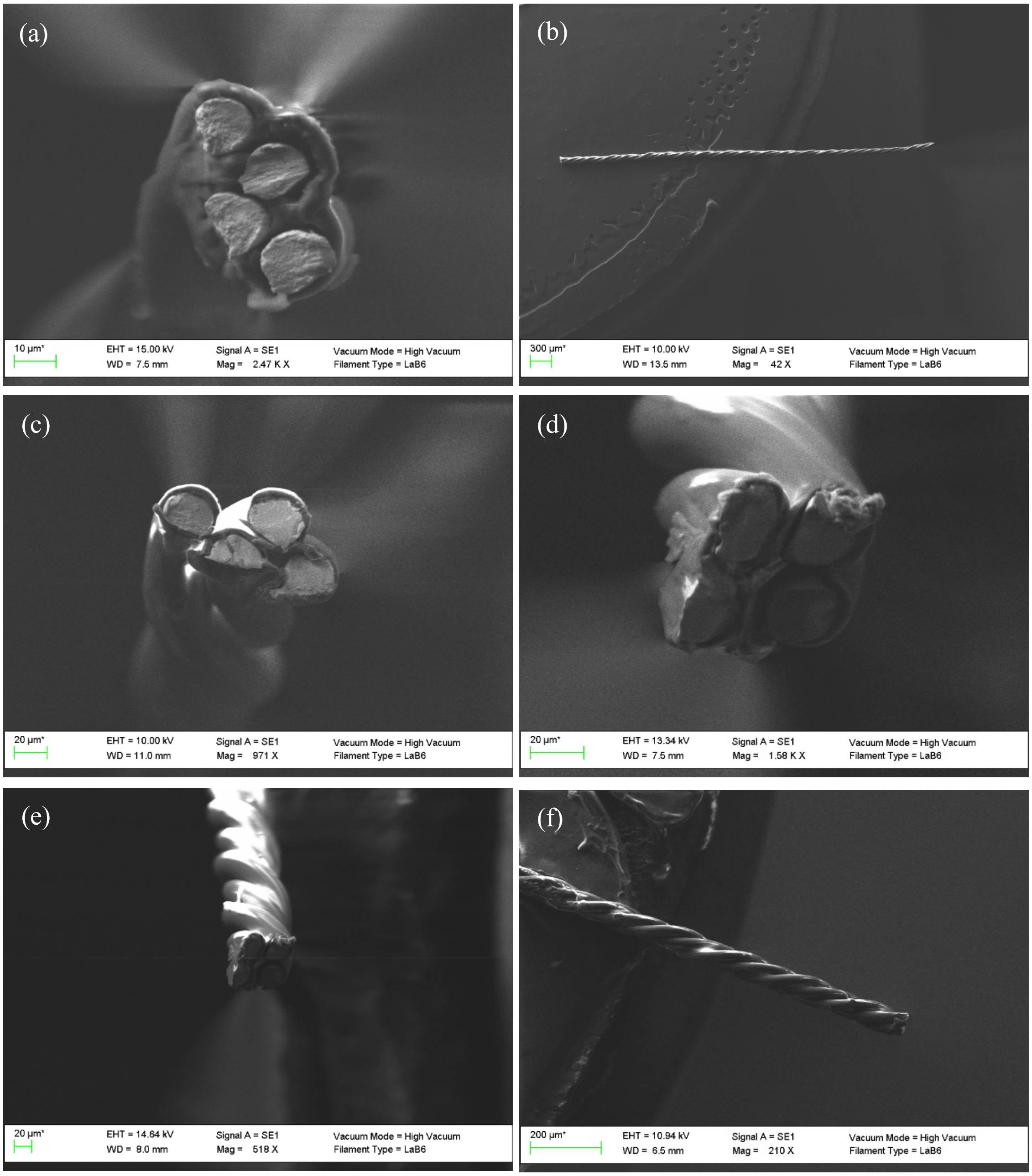

Figure S9: (a) W tetrode with heat curing at 200˚C. The outer insulation (HFV) is fused together, and the four wires are closely packed. **(b,c)** Pt tetrode without heat curing, body, and cross-section images. Four wires split at the end of the tetrode. **(d~f)** Pt tetrode with heat curing at 300 ˚C, cross-section, and body images. The Pt wires are closely packed.

2.3.2 Cross-connection check

These checks were made to ensure that the electrodes within a tetrode were electrically insulated from one another.

Experimental procedures

Electrochemical Method: Initially, the Pt tetrode was immersed in a solution containing 0.1 mM NaPSS. Three successive CV scans from OCP to 1.5 V (vs. SCE) and back to OCP were conducted for two wires on the tetrode, respectively, designated as Wire 1 and Wire 2. Wire 1 was selected for coating, while Wire 2 was to remain bare, and their CV scans would be compared once again in 0.1mM NaPSS after coating. The tetrode was then placed in a solution containing 10 mM EDOT and 0.1 mM NaPSS. Wire 1 underwent galvanostatic deposition at 20 nA for 13 s. The wire was then gently dipped into deionized water to wash away any excess monomer solution from the surface before being returned to the NaPSS solution for a post-coating test. The same CV sweep conducted prior to coating was repeated, allowing the results to be overlaid and compared.

To test for unwanted cross-connections between different Pt wires in the tetrode after coating, the tetrode was then immersed in 0.1 mM NaPSS and a CV scan was performed for each wire within the same potential window as before (OCP → 1.5 V → OCP). If any cross-connection

exists, one CV scan in NaPSS will disrupt the polymer electroactivity on any connected wire, thus eliminating the overoxidation peak. If the process is successful, four distinct oxidation peaks should be evident during the post-coating scans, one for each electrode contacted.

Spectroscopic analysis: For more direct visual evidence, deposition was performed on three of the four discs. The resulting tetrode was then cut and meticulously affixed to a specimen stub for SEM and EDX analysis. Additionally, the tetrode segment could be examined under an optical microscope to assess the coating. In both cases, the coated end must be delicately handled and oriented toward the camera for clear visualization, enabling the coated cross-section of the tetrode to be clearly seen. This allows for a direct comparison with the cross-section of the bare tetrode.

Results:

Electrochemical Method: CV scans of a coated (Wire 1) and an adjacent bare wire (Wire 2) in 0.1mM NaPSS were compared before and after polymer deposition. As illustrated in Figure S10(a), after Wire 1 was coated with PEDOT:PSS, a significant peak corresponding to polymer overoxidation is evident in the first CV scan cycle. In the subsequent cycles, the scans align resemble those seen before coating, indicating that polymer electroactivity was destroyed in the first scan, rendering the response indistinguishable from that of bare Pt. The CV scans of the uncoated Wire 2, depicted in Figure S10(b), show minimal difference before and after the coating of Wire 1. This demonstrates that individual tetrode discs can be coated without affecting neighboring discs. The slight increase observed in Wire 2 (at ca 1.0 V, Figure S10(b)) after coating Wire 1 is attributed to EDOT adsorption rather than overgrowth. Otherwise, scanning Wire 1 would have completely eliminated any peaks in Wire 2.

Subsequently, all wires were coated in sequence (noted as Wires 1 to 4). Their galvanostatic deposition curves, presented in Figure S10(c), show a relatively consistent initiation point, indicating that the polymerization commenced on the Pt substrate without any previous potential overgrowth. All four wires exhibit overoxidation peaks in the first cycle of their CV scans in NaPSS (Figure S10(d)), and the subsequent cycles (Figure S10(e)) confirmed that all polymer-associated electroactivity was eradicated. These findings confirm that the destruction of polymer on one tetrode disc did not impact the others, suggesting that the coatings are independent on each wire without any electrical cross-connection. Moreover, the uniformity in the height and shape of all four overoxidation peaks (Figure S10(d)) suggests that a consistent level of deposition was achieved.

Spectroscopic analysis: Three of the four wires on the tetrode were coated, and their characterizations are presented in Figure S11. It is challenging to determine from the SEM image alone (Figure S11(a)) whether the surface is covered with PEDOT. However, EDX provides insights into the elements present on the surface (Figure S11(d)). The EDX spectra of the coated discs, as shown in Figure S11(d), revealed the presence of carbon (C) and oxygen (O) elements, while the bare and partially exposed electrodes exhibited significant peaks for platinum (Pt). The high levels of aluminum (Al) observed originate from the sample stage. Optical microscopy offered more direct visual evidence; the bare Pt appears shiny, whereas the PEDOT-covered surfaces are dull (Figures S11(b, c)). Additionally, Figures S11(b, c) clearly show that the PEDOT adhered well to the plates without encroaching onto the other discs.

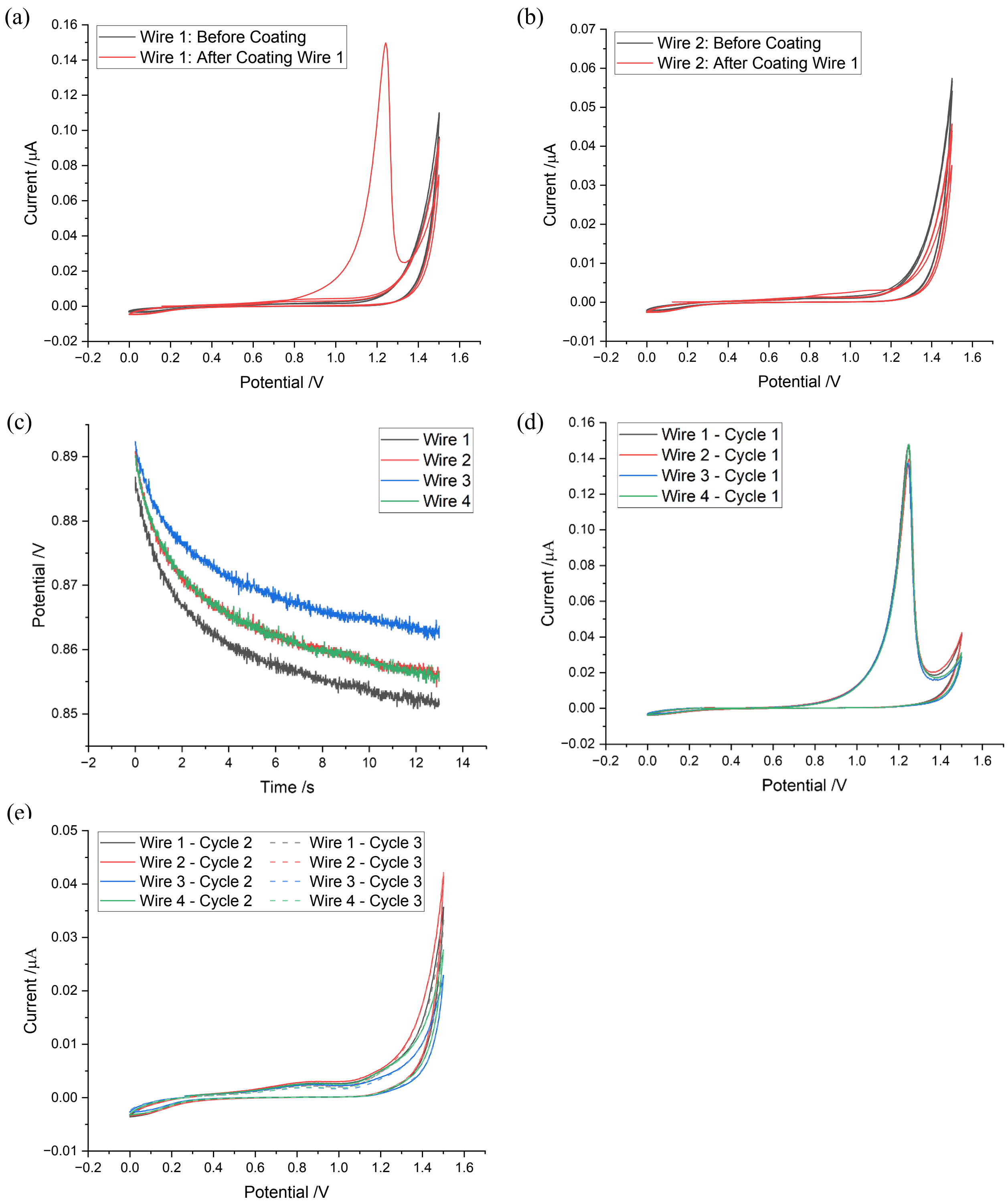

Figure S10: (a) CV scan of wire 1 before and after coating in 0.1mM NaPSS (OCP → 1.5 V →OCP). **(b)** CV scan in 0.1 mM NaPSS of wire 2 before and after coating wire 1 (OCP → 1.5 V

→OCP). (The other bare wires had similar results as Wire 2). **(c)** Galvanostatic deposition of all four wires at 20 nA for 13 s. **(d, e)** The CV scan for all four wires in 0.1 mM NaPSS (OCP → 1.5 V →OCP). **(d)** The first cycle of the CV. Overoxidation peaks can be observed for all wires. **(e)** Second (solid lines) and third cycles (dashed lines) of CV scan for all four wires. Scan rate v = 50 mVs^{-1} for all the above CV scans. All potentials are reported relative to the SCE.

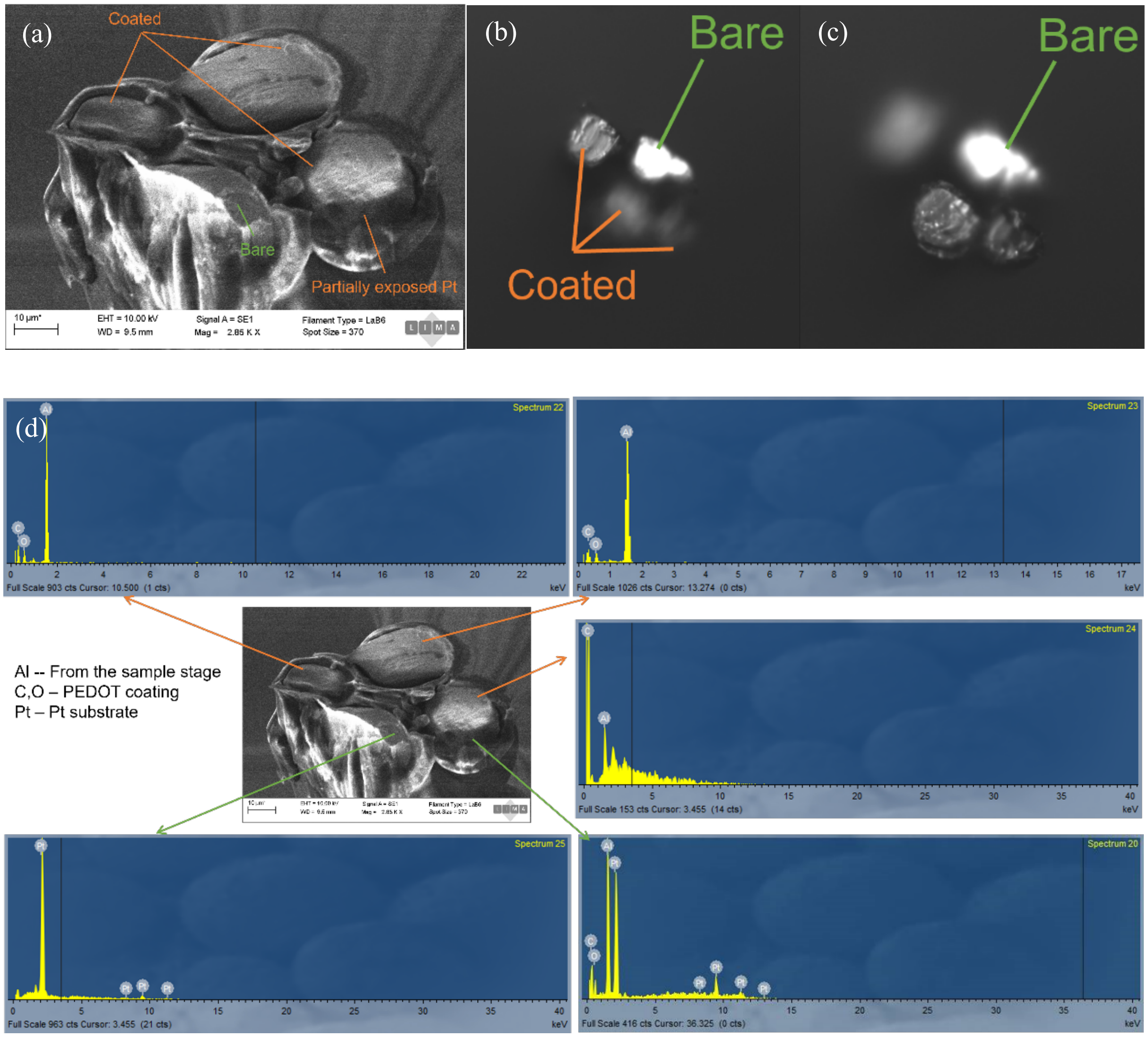

Figure S11: (a) SEM image of partially coated Pt tetrode. **(b,c)** Optical microscope image of partially coated Pt tetrode. **(d)** EDX for analyzing elements on the surface as shown in (a).

Section 3: Potential Step Experiments

This section includes details of the experiment procedure, CV scans of the polymer-coated electrode to identify the unreactive region, data smoothing, and details of the potential step experiment and analysis.

3.1 Identifying Faradaically Unreactive Regions

Prior to initiating the stepped potential experiment, so as to identify start and end potentials for the steps, the signal-receiving electrode (WE2) was subjected to a series of CV scans extending from the open circuit potential (OCP) to various maximum potentials (E_{max}). The outcomes for the bare Pt are discussed in the main paper (Section 3.2 Figure 4(a)), and the following pictures (Figure S12) are the CV scans for the PEDOT-coated electrodes.

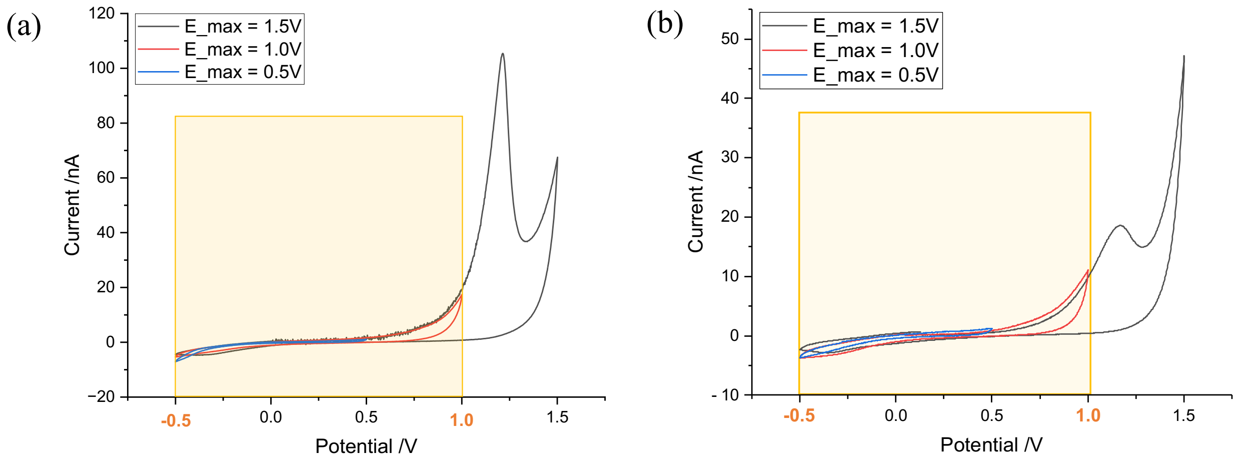

Figure S12: CV scan from OCP → E_{max} → -0.5 V was employed using (scan rate v = 50 mVs^{-1}). **(a)** PEDOT:PSS coated, **(b)** PEDOT:Cl coated microwire immersed in 0.01 M PBS. For both polymers, -0.5V to 1.0V is a safe region without triggering any Faradaic current. All potentials are reported relative to the SCE.

3.2 PEDOT Coated Potential Step Graphs and Data Smoothing

3.2.1 Data Smoothing

For electrodes coated with PEDOT, recorded currents demonstrate greater variability than those from bare Pt electrodes. The raw transients have significant noise, making overlaying and comparison challenging. Consequently, data were smoothed using MATLAB. The smoothing parameter ($p_s = 0.999991$, hereafter referred to as p_i) was tuned to preserve the prominent data features while enhancing presentation clarity. Examples of the smoothed data are depicted in Figures S13 (a~d), using data from a PEDOT:PSS coated electrode. Post-smoothing, the data from the PEDOT:PSS coated WE2 are much clearer, maintaining crucial information preceding and following the application of the potential step (Figures S13(a, b)), thus simplifying comparison with a bare Pt WE2 (Figures S13(c, d)). Although PEDOT:Cl shows less variation than PEDOT:PSS, smoothing was also found to benefit the data analysis (Figures S13 (e, f)).

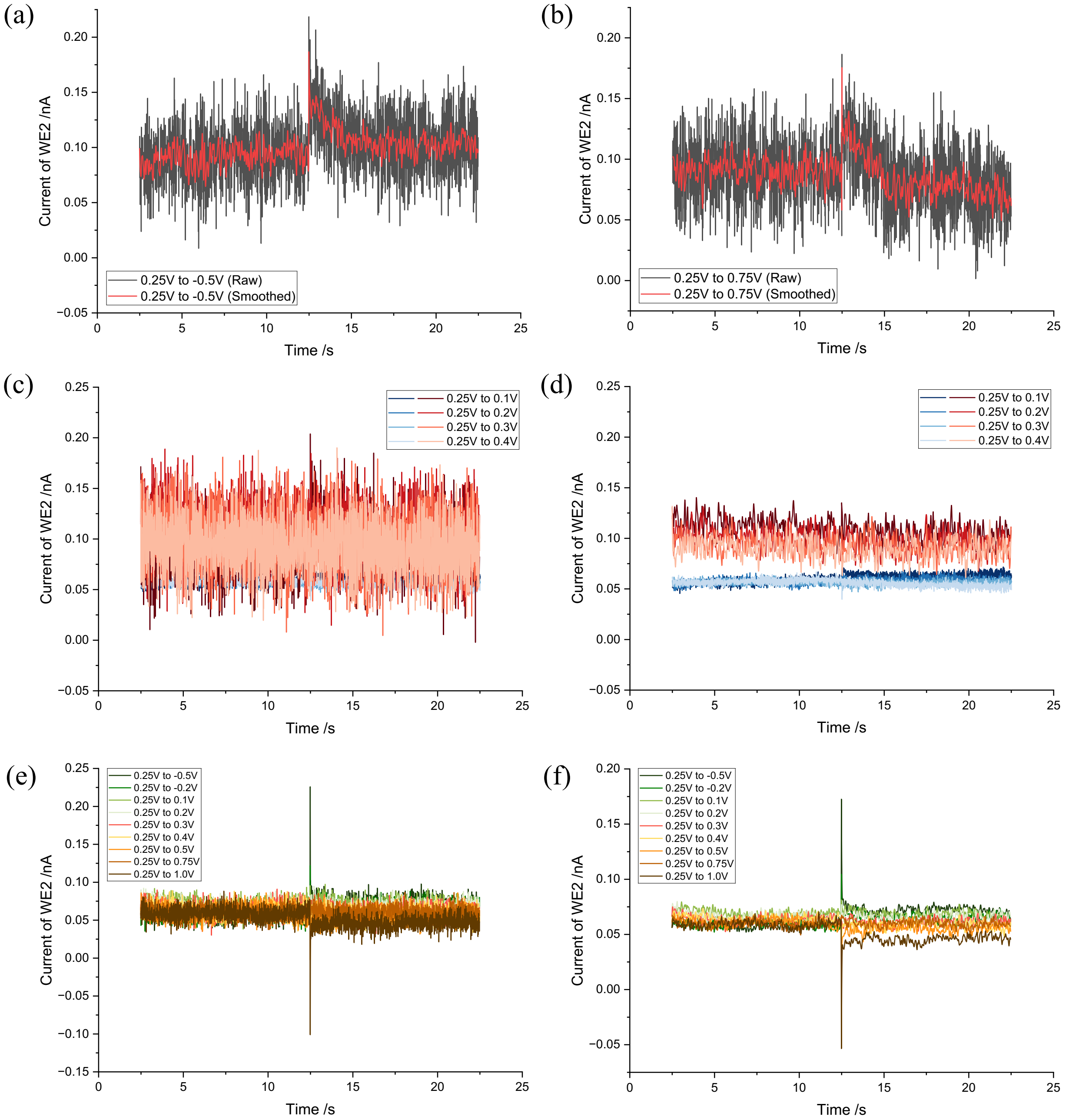

Figure S13: (a, b) Comparison of the raw (black line) and smoothed (red line) data of PEDOT:PSS coated WE2 (WE2 held at 0.25 V). **(c, d)** Comparison between the bare (blue lines) and PEDOT:PSS (red lines) coated WE2 recorded data (WE2 held at 0.25 V), where PEDOT:PSS data was unprocessed in (c) and smoothed in (d). **(e, f)** PEDOT:Cl potential step data (WE2 held at 0.25 V), where (e) is the unprocessed raw data and (f) is smoothed. All potentials are reported relative to the SCE.

3.2.2 PEDOT:Cl Potential Step Experiment Figures

Figures S14 presents the results of the potential step experiment following Scheme 2 in the main paper, using a tetrode with WE2 coated with PEDOT:Cl and immersed in 0.01 M PBS.

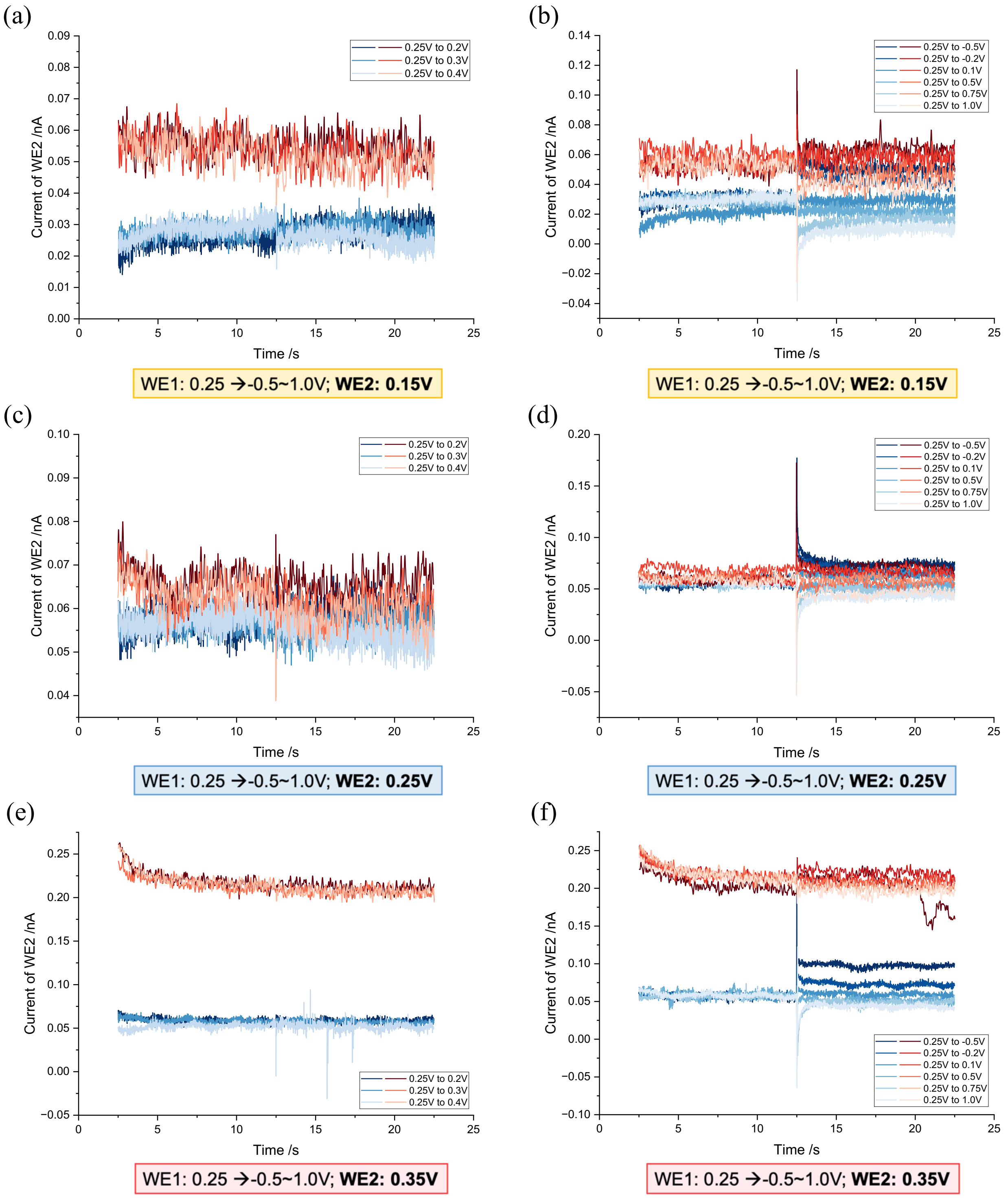

Figure S14: The currents recorded on WE2, where WE2 was held at 0.25 V/0.15 V/0.35 V, respectively, when the potential on WE1 jumped from 0.25 V to a range of final potentials. The left column graphs (a, c, e) illustrate the situations where the potential step on WE1 triggered little response on WE2. The right column graphs (b, d, f) show the potential steps that led to significant responses on WE2. *Blue lines:* Bare Pt. *Red lines:* PEDOT:Cl coated Pt. All potentials are reported relative to the SCE.

3.3 Potential Step Data Analysis and Preprocessing

This section presents the method of fitting the current transient recorded at WE2 following a potential step applied to WE1 with WE2 held at a fixed potential using the following equation:

$$I = I_0 + A_1 exp\left(-\frac{t}{t_1}\right) \tag{11}$$

I_0 denotes the steady-state current; t_1 is the response time, reflecting how ions around WE2 respond to the sudden potential change at WE1; the sign of A_1 indicates the direction of the resulting current after the potential step, where a positive A_1 suggests a positive current, and vice versa. It needs to be noted that the fitting of equation 11 focuses on the transient decay after the initial rapid spike (> 0.02 s). Additionally, to avoid tiny current pulses and to better fit the exponential curves, large potential steps on WE1 were chosen to obtain significant responses. Specifically, V_f was chosen to satisfy $| V_f - V_i | > 0.35$ V, with $V_i = 0.25$ V (Table S2). The procedures were repeated for WE2 held at 0.15 V, 0.25 V, and 0.35 V. The data fitting was performed using the software Origin 2024.

Noting the noisy nature of the recorded current and the approximation of applying equation (11), the R-square value for fitting was generally controlled to be around 0.5. However, for very noisier

data, notably WE2 coated with a polymer film, the variance of the raw data is much higher, causing the R-square for fitting the original data to be lower. Hence, in this case, both the raw data and its smoothed data were fitted with equation 11, with the latter one to double-check the validity of the fitting.

Table S2: The potential step experiments for WE1 when WE2 is a PEDOT-coated electrode. All potentials are reported relative to the SCE.

WE1 Initial Potential, V_i (V)	WE1 Final Potential, V_f (V)	Potential Step Size, $V_f - V_i$ (V)
0.25	-0.5	-0.75
	-0.35	-0.6
	-0.25	-0.5
	-0.2	-0.45
	-0.1	-0.35
	0.6	0.35
	0.7	0.45
	0.75	0.5
	0.85	0.6
	1.0	0.75

3.3.1 Bare Pt Electrode

Figure S15 presents a summary of the data preprocessing for the bare Pt WE2 electrode. Given that the current typically returns to a steady state approximately 2 s after a change in potential on WE1, extending the data fitting to the full 10 s is unnecessary and shows minimal difference from a 4 s data fit (Figures S15(a, b)). The fitting parameters (I_0, t_1 , A_1) for both the 10 s and 4 s datasets closely align, as indicated in the tables below the graphs, with minor variances falling about the error margins of each other.

The low R-Square value originates from the inherent noise in the recorded signals. To improve the quality and check the reliability of the fitting, data were smoothed using MATLAB with a smoothing parameter of $p_s = 0.9999$ (Figure S15(c)), followed by fitting with equation 11 in Origin. As shown in the table in Figure S15(d), the R-Square value improved to 0.864, and all fitting parameters remained consistent with those obtained from the raw data fitting (Figure S15(b)). For example, the response time of the smoothed data ($t_1^{smoothed} \approx 0.22 \pm 0.01\, s$) aligns with the response time derived from raw data ($t_1^{raw} \approx 0.24 \pm 0.04\, s$). Furthermore, some datasets exhibit minimal fluctuations (Figure S16(d, e)), thus their fitting results do not necessitate additional validation through smoothing.

(a)

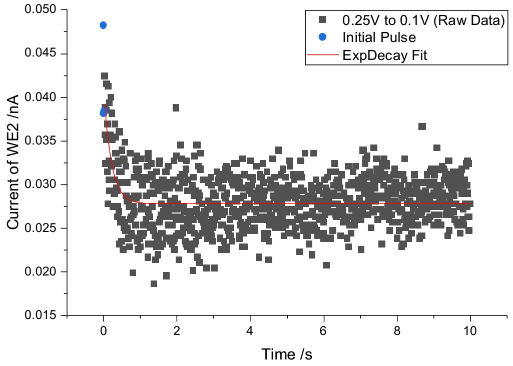

Model	ExpDec1
Equation	I = A1*exp(-x/t1) + I0
Plot	Current of WE2
I0	0.02782 ± 9.39988E-5
A1	0.01322 ± 0.00159
t1	0.20791 ± 0.03197
Reduced Chi-Sqr	8.07431E-6
R-Square (COD)	0.14575
Adj. R-Square	0.14403

(b)

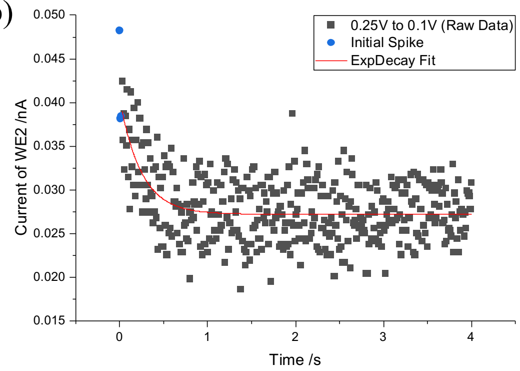

Model	ExpDec1
Equation	I = A1*exp(-x/t1) + I0
Plot	Current of WE2
I0	0.02724 ± 1.94078E-4
A1	0.01324 ± 0.00168
t1	0.2433 ± 0.04238
Reduced Chi-Sqr	1.1325E-5
R-Square (COD)	0.25407
Adj. R-Square	0.25029

(c)

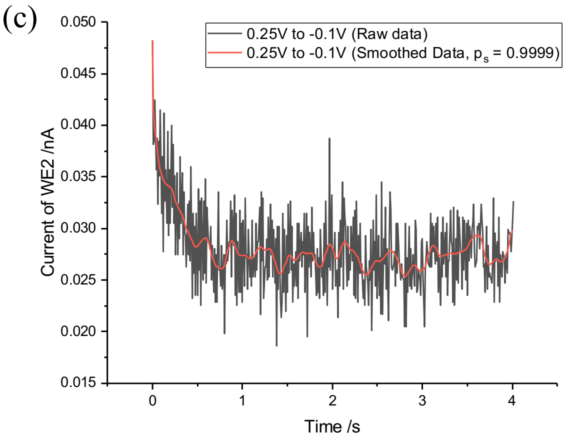

(d)

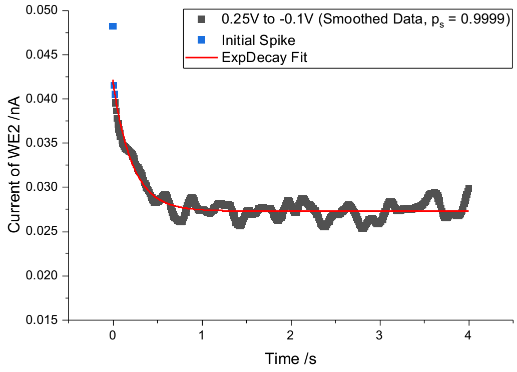

Model	ExpDec1
Equation	I = A1*exp(-x/t1) + I0
Plot	Current of WE2
I0	0.02727 ± 5.30227E-5
A1	0.01487 ± 3.86708E-4
t1	0.21599 ± 0.00867
Reduced Chi-Sqr	8.84438E-7
R-Square (COD)	0.8637
Adj. R-Square	0.86301

(e)

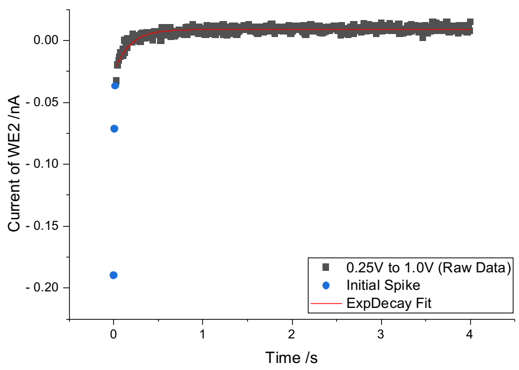

Model	ExpDec1
Equation	I = A1*exp(-x/t1) + I0
Plot	Current of WE2
I0	0.00876 ± 1.34585E-4
A1	-0.03497 ± 0.00157
t1	0.17326 ± 0.00999
Reduced Chi-Sqr	5.9533E-6
R-Square (COD)	0.75492
Adj. R-Square	0.75368

(f)

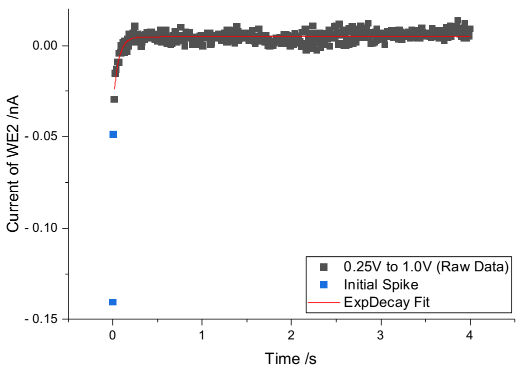

Model	ExpDec1
Equation	I = A1*exp(-x/t1) + I0
Plot	Current of WE2
I0	0.00478 ± 1.43635E-4
A1	-0.04052 ± 0.00392
t1	0.05765 ± 0.00619
Reduced Chi-Sqr	7.75484E-6
R-Square (COD)	0.46955
Adj. R-Square	0.46687

Figure S15: (a, b) 10 s and 4 s data for currents recorded at WE2 following a potential step at WE1 from 0.25 V to -0.1 V. WE2 was held at 0.25 V. **(c)** Comparison of the smoothed data and the raw data. The smoothing parameter $p_s = 0.9999$. **(d)** Curve fitting of the smoothed data in (c). The fitting parameters (I_0, t_1 and A_1) are all within the error range of that presented in the table in (b). **(e, f)** Less noisy data. WE2 was held at (e) 0.25 V, (f) 0.35 V. All potentials are reported relative to the SCE.

3.3.2 PEDOT:PSS Coated Electrodes

In contrast to bare Pt, PEDOT:PSS coated electrodes exhibited a longer response time, hence the full dataset of 10 s was used for fitting. Figure S16 provides a fitting of the current measured at WE2 (held at 0.15 V) after WE1 potential jumped from 0.25 V to -0.1 V. Initially using the same smoothing parameter as in section 3.2.1 ($p_s = p_i$), the fit yielded an R-square of 0.341 (Figure S16(a)). A stronger smoothing on the raw data was therefore performed to check the reliability. The R-square value increased to 0.486 when $p_s = 0.9999$ is set (Figure S16(b)), and the fitting parameters are well within the error range of the ones in Figure S16(a). Care was taken to avoid over-smoothing and associated loss of data integrity.

(a)

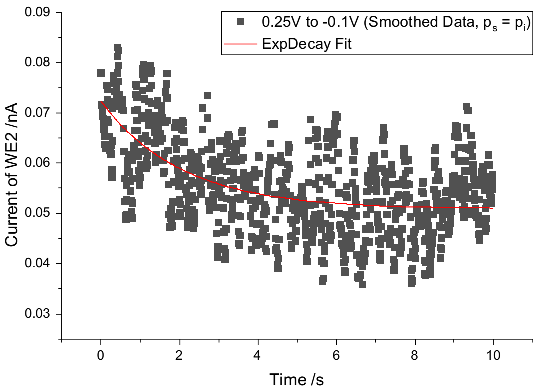

Model	ExpDec1
Equation	I = A1*exp(-x/t1) + I0
Plot	Current of WE2
I0	0.05082 ± 4.81782E-4
A1	0.02141 ± 0.00105
t1	2.05532 ± 0.22385
Reduced Chi-Sqr	5.60044E-5
R-Square (COD)	0.34113
Adj. R-Square	0.33975

(b)

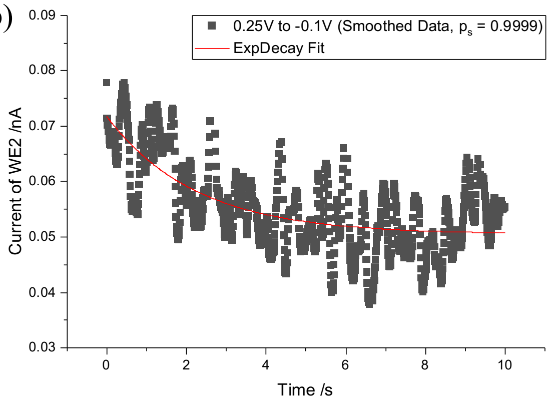

Model	ExpDec1
Equation	I = A1*exp(-x/t1) + I0
Plot	Current of WE2
I0	0.0505 ± 3.94447E-4
A1	0.02126 ± 7.56106E-4
t1	2.23474 ± 0.18666
Reduced Chi-Sqr	3.07791E-5
R-Square (COD)	0.48608
Adj. R-Square	0.485

Figure S16: The current recorded on WE2, when WE2 was held at 0.15 V, respectively, and the potential on WE1 jumped from 0.25 V to -0.1 V. **(a)** The data was preprocessed with a smoothing parameter $p_s = p_i = 0.999991$ **(b)** $p_s = 0.9999$. All potentials are reported relative to the SCE.

3.3.3 PEDOT:Cl Coated Electrodes

In the case of PEDOT:Cl-coated WE2s, the response to potential changes on the adjacent electrode is fast (< 0.1 s). Therefore, the fitting was performed using data from the initial 4 s instead of the entire 10 s recorded (Figure S17).

As the smoothing level was progressively increased, using $p_s = p_i$, 0.9999, and 0.999, the R-Square value improved from 0.105 to 0.242, and lastly to 0.718 (Figure S17). Moreover, the fitting parameters derived from these varying levels of smoothing all fall within the margin of error for the least smoothed data (ps = pi). Specifically, for $p_s = 0.9999$ and 0.999, $t_1 = 0.029 \pm 0.005\, s$

and $0.025 \pm 0.002\ s$, respectively. These values are within the error range of $t_1 = 0.023 \pm 0.007\ s$ obtained with the gentlest smoothing $p_s = p_i$.

(a)

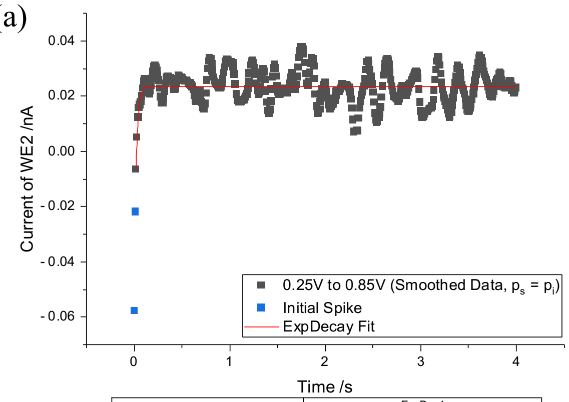

Model	ExpDec1
Equation	I = A1*exp(-x/t1) + I0
Plot	Current of WE2
I0	0.02353 ± 2.86937E-4
A1	-0.07173 ± 0.02817
t1	0.02251 ± 0.00687
Reduced Chi-Sqr	3.18503E-5
R-Square (COD)	0.10457
Adj. R-Square	0.10001

(b)

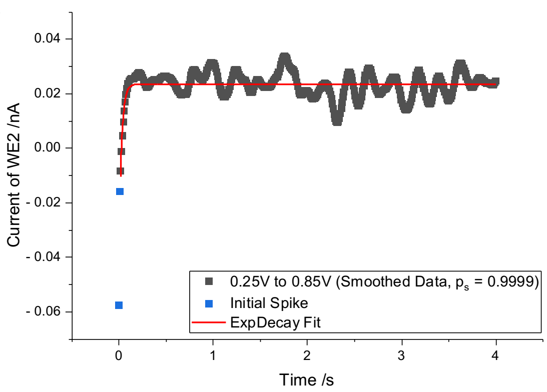

Model	ExpDec1
Equation	I = A1*exp(-x/t1) + I0
Plot	Current of WE2
I0	0.02342 ± 2.14858E-4
A1	-0.0682 ± 0.01422
t1	0.02863 ± 0.0052
Reduced Chi-Sqr	1.78853E-5
R-Square (COD)	0.24152
Adj. R-Square	0.23769

(c)

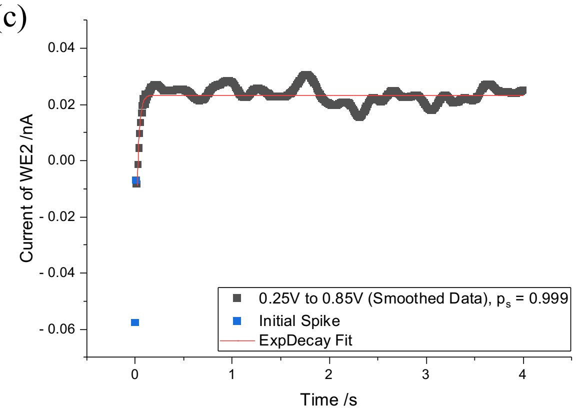

Model	ExpDec1
Equation	I = A1*exp(-x/t1) + I0
Plot	Current of WE2
I0	0.02341 ± 1.52664E-4
A1	-0.07195 ± 0.0027
t1	0.02484 ± 0.0016
Reduced Chi-Sqr	9.1111E-6
R-Square (COD)	0.71824
Adj. R-Square	0.71682

Figure S17: The current recorded on WE2, where WE2 was held at 0.35 V, respectively, and the potential on WE1 jumped from 0.25 V to 0.85 V. **(a)** The data was preprocessed with a smoothing parameter $p_s = p_i = 0.999991$ **(b)** $p_s = 0.9999$. **(c)** $p_s = 0.999$. All potentials are reported relative to the SCE.

Section 4: In Vivo Experiment

A pilot study has been conducted to test the biocompatibility of the PEDOT-coated tetrode. Detailed experimental procedures and data collection are included in this section. In the end, a primary analysis was conducted to compare the recording quality of PEDOT-coated electrodes.

4.1 Materials and Methods

Surgical Procedure: One adult mouse was involved in the pilot study. Experimental procedures were carried out in strict accordance with the Animals (Scientific Procedures) Act, 1986 (United Kingdom), with a final ethical review by the Animals in Science Regulation Unit of the UK Home Office. All surgical procedures were performed under deep anesthesia using isoflurane (0.5–2%) and oxygen (2 l/min), with analgesia provided before (0.1 mg/kg vetergesic) and after (5 mg/kg metacam) surgery [15, 16]. For the in vivo recording, the mouse was implanted with a single microdrive containing 14 independently vertically adjustable tetrodes (4 W tetrodes, 3 Pt tetrodes, 4 PEDOT:PSS coated Pt tetrodes, 3 PEDOT:Cl coated Pt tetrodes. Tetrode fabrication and polymer deposition procedures are introduced in section 3). Each tetrode was loaded into a cannula on the microdrive. Every cannula is connected to a screw (M.10, length = 6mm,) to allow independent control of a tetrode's depth. The target layer was the CA1 region of the hippocampus. After implantation, the exposed parts of the tetrodes were sealed and covered with paraffin wax. Subsequently, the drive was affixed to the skull utilizing dental cement and stainless-steel anchor screws. Two of these anchor screws, both positioned above the cerebellum, were connected to a 50 µm W wire (California Fine Wire) and served to provide a ground potential [15, 16] .For the recordings, each tetrode was carefully adjusted downwards to target the CA1 pyramidale layer of

the hippocampus. This adjustment was made by turning a screw connected to each cannula that controls the tetrode's position. The correct placement of tetrode is determined by observing the electrical signals, specifically the electrophysiological profile of the local field potentials in the hippocampal ripple frequency band, which is characteristic of this brain region [15, 16].

Recording Procedure and Data Acquisition: Following full recovery from surgery, signal recording started by the end of the fifth week after the surgery. Before starting the recordings on the day, the positions of the tetrodes were finely adjusted to achieve optimal clarity and quantity of spike waveforms, as determined by visual inspection. The extracellular signals from each recording channel were then amplified, combined, and digitized using a single integrated circuit on the head of the animal (RHD2164, Intan Technologies; http://intantech.com/products_RHD2000.html; pass band 0.09 Hz to 7.60 kHz) [15, 16]. These processed signals were digitized at a rate of 20 kHz and subsequently stored on a disk.

Spike detection and unit isolation: Spike sorting and unit isolation were conducted using an automated clustering pipeline implemented in Kilosort through the SpikeForest framework [15-18]. When processing data acquired from tetrodes, Kilosort confines its templates to channels within a specified tetrode bundle and excludes all other recording channels. The operator then verified the resulting clusters by analyzing cross-channel spike waveforms and examining both auto-correlation and cross-correlation histograms.

4.2 Preliminary analysis of the recording

As mentioned in the manuscript section 4, a waveform score (wvscore) has been introduced to compare the quality of the recording:

$$wv_{score} = \sqrt{\sum_{i=1}^{n} \frac{(w_i/\sigma_{wi})^2}{n}} \tag{12}$$

where w_i is the value of mean waveform of a sample i, σ_{wi} is the standard deviation across all spike of sample i, and *n* is the number of waveform samples [16]. Figure S18 (a, b) presents examples of low and high wvscore, respectively. To understand these figures, it can be considered as how the mean waveform (represented by the thick, darker line) compares to the distribution of all waveforms (illustrated by the lighter background band). Figure S18(b) shows a narrower band, suggesting a smaller deviation, which could indicate less noise and more consistent recordings over time. In contrast, Figure S18(a) displays a wider background band relative to the mean waveform, suggesting greater variation during recording and reduced consistency.

Furthermore, wvscores were compared between electrodes coated with PEDOT:PSS and PEDOT:Cl (Figure S19). It appears that PEDOT:Cl provided a better quality of recording, as the distribution of the wvscores favors the high-value side. However, given the nature of the in vivo experiments, drawing more definitive conclusions requires further studies. Nonetheless, this pilot study has established a solid foundation for future investigation.

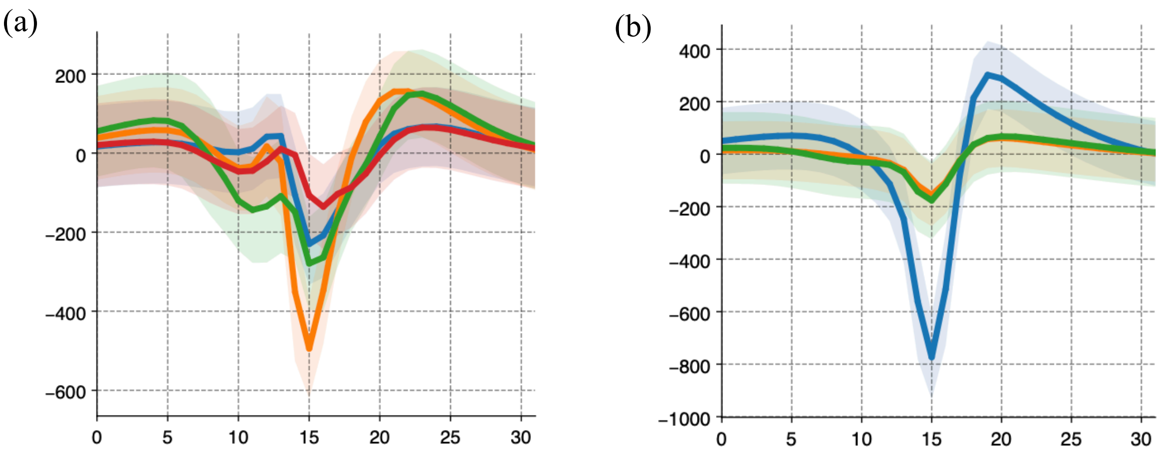

Figure S18: Example of **(a)** low wvscore (0.64) and **(b)** high wvscore (1.44).

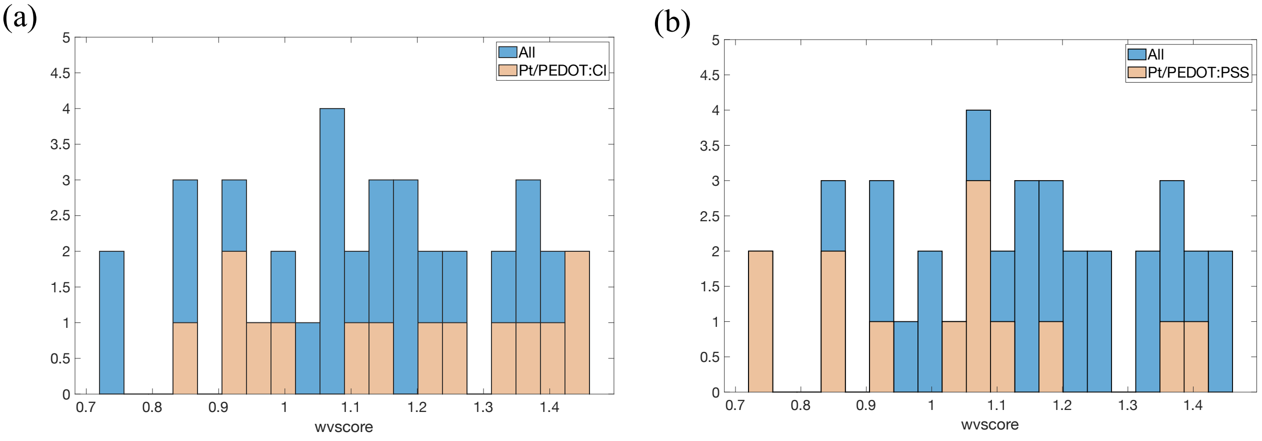

Figure S19: Histogram of all the wvscores obtained from single-unit recording. The blue bins represent the wvscore from all the recordings. The pale orange bins represent the wvscore obtained with **(a)** PEDOT:Cl coated tetrode; **(b)** PEDOT:PSS coated tetrode.

References:

(1) Zhang, Y.; Chen, Y.; Contera, S.; Compton, R. G. Electrochemical and Nanostructural Characterization of Poly(3,4-ethylenedioxythiophene):poly(styrenesulfonate) Films as Coatings for Neural Electrodes. *ACS Applied Polymer Materials* **2023**, *5* (7), 5555-5566.

(2) Patil, R. S.; Juvekar, V. A.; Naik, V. M. Oxidation of Chloride Ion on Platinum Electrode: Dynamics of Electrode Passivation and its Effect on Oxidation Kinetics. *Industrial & Engineering Chemistry Research* **2011**, *50* (23), 12946-12959.

(3) Puglia, M. K.; Bowen, P. K. Cyclic Voltammetry Study of Noble Metals and Their Alloys for Use in Implantable Electrodes. *ACS Omega* **2022**, *7* (38), 34200-34212.

(4) Dickinson, T.; Greef, R.; Wynne-Jones, L. The kinetics of the chlorine electrode reaction at a platinum electrode. *Electrochimica Acta* **1969**, *14* (6), 467-489.

(5) Chen, Y.; Compton, R. G. Direct Electrochemical Analysis in Seawater: Evaluation of Chloride and Bromide Detection. *Chemosensors* **2023**, *11* (5), 297.

(6) Geddes, L. A.; Roeder, R. Criteria for the selection of materials for implanted electrodes. *Ann Biomed Eng* **2003**, *31* (7), 879-890.

(7) Williams, J. C.; Rennaker, R. L.; Kipke, D. R. Long-term neural recording characteristics of wire microelectrode arrays implanted in cerebral cortex. *Brain Research Protocols* **1999**, *4* (3), 303-313.

(8) Patrick, E.; Orazem, M. E.; Sanchez, J. C.; Nishida, T. Corrosion of tungsten microelectrodes used in neural recording applications. *Journal of Neuroscience Methods* **2011**, *198* (2), 158-171.

(9) Kelsey, G. S. The anodic oxidation of tungsten in aqueous base. *Journal of the Electrochemical Society* **1977**, *124* (6), 814.

(10) Lillard, R.; Kanner, G.; Butt, D. The nature of oxide films on tungsten in acidic and alkaline solutions. *Journal of the Electrochemical Society* **1998**, *145* (8), 2718.

(11) Tamburri, E.; Orlanducci, S.; Toschi, F.; Terranova, M. L.; Passeri, D. Growth mechanisms, morphology, and electroactivity of PEDOT layers produced by electrochemical routes in aqueous medium. *Synthetic Metals* **2009**, *159* (5-6), 406-414.

(12) Tamburri, E.; Orlanducci, S.; Toschi, F.; Terranova, M. L.; Passeri, D. Growth mechanisms, morphology, and electroactivity of PEDOT layers produced by electrochemical routes in aqueous medium. *Synthetic Metals* **2009**, *159* (5), 406-414.

(13) Du, X.; Wang, Z. Effects of polymerization potential on the properties of electrosynthesized PEDOT films. *Electrochimica Acta* **2003**, *48* (12), 1713-1717.
(14) *Enamel Insulation Specifications*. California Fine Wire, https://calfinewire.com/wp-content/uploads/Enamel-Insulation-Specs.pdf (accessed 2024 02.16).
(15) Van de Ven, G. M.; Trouche, S.; McNamara, C. G.; Allen, K.; Dupret, D. Hippocampal offline reactivation consolidates recently formed cell assembly patterns during sharp wave-ripples. *Neuron* **2016**, *92* (5), 968-974.
(16) Vítor, L.-d.-S.; Demi, B.; David, D. Spatio-temporal organization of network activity patterns in the hippocampus. *bioRxiv* **2023**, 2023.2010.2017.562689.
(17) Magland, J.; Jun, J. J.; Lovero, E.; Morley, A. J.; Hurwitz, C. L.; Buccino, A. P.; Garcia, S.; Barnett, A. H. SpikeForest, reproducible web-facing ground-truth validation of automated neural spike sorters. *eLife* **2020**, *9*, e55167.
(18) Pachitariu, M.; Steinmetz, N. A.; Kadir, S. N.; Carandini, M.; Harris, K. D. Fast and accurate spike sorting of high-channel count probes with KiloSort. *Advances in neural information processing systems* **2016**, *29*.
